# Comprehensive data on a 2D-QSAR model for Heme Oxygenase isoform 1 inhibitors

**DOI:** 10.1016/j.dib.2017.09.036

**Published:** 2017-09-21

**Authors:** Emanuele Amata, Agostino Marrazzo, Maria Dichiara, Maria N. Modica, Loredana Salerno, Orazio Prezzavento, Giovanni Nastasi, Antonio Rescifina, Giuseppe Romeo, Valeria Pittalà

**Affiliations:** a Department of Drug Sciences, University of Catania, Viale A. Doria 6, 95125 Catania, Italy; b Department of Mathematics and Computer Sciences, University of Catania, Viale A. Doria 6, 95125 Catania, Italy

**Keywords:** Heme Oxygenase, 2D-QSAR, pIC50 prediction, FDA, CORAL

## Abstract

The data have been obtained from the Heme Oxygenase Database (HemeOxDB) and refined according to the 2D-QSAR requirements. These data provide information about a set of more than 380 Heme Oxygenase-1 (HO-1) inhibitors. The development of the 2D-QSAR model has been undertaken with the use of CORAL software using SMILES, molecular graphs and hybrid descriptors (SMILES and graph together). The 2D-QSAR model regressions for HO-1 half maximal inhibitory concentration (IC_50_) expressed as pIC_50_ (pIC_50_=−LogIC_50_) are here included. The 2D-QSAR model was also employed to predict the HO-1 pIC_50_values of the FDA approved drugs that are herewith reported.

**Specifications Table**

**Table:** 

Subject area	Computational Chemistry
More specific subject area	Quantitative Structure-Activity Relationship (QSAR) modeling
Type of data	Table, figure
How data was acquired	Statistical modeling and online databases
Data format	Raw and analyzed
Experimental factors	The whole dataset consists of 382 HO-1 inhibitors which were randomly split and divided into training, invisible training, calibration, and validation sets.
Experimental features	The 2D-QSAR models have been developed using CORAL software. Chemical structure descriptors and pIC_50_ were used as variables.
Data source location	Department of Drug Sciences, Department of Mathematics and Computer Sciences, University of Catania, Italy
Data accessibility	With this article

**Value of the data**

• HO-1 is a crucial enzyme involved in the catabolism of heme and overexpressed in a number of tumors with poor clinical outcome.
• 2D-QSAR modeling data was generated to provide a method useful in finding or repurposing novel HO-1 inhibitors.
• The model has also been used to predict the HO-1 pIC_50_ for the FDA-approved drugs.

## 1 Data

HO-1 is a crucial enzyme involved in the regioselective catabolism of heme. Strongly induced upon stressful condition, HO-1 is recognized to fulfil crucial roles in cytoprotection and in the maintenance of endogenous homeostasis, playing a role in metabolic, cardiovascular, and pulmonary diseases [1], [2], [3]. Nevertheless, under adverse circumstances it has been demonstrated that aberrant levels of HO-1 may sustain cancerous diseases. Therefore, its inhibition is of interest in all such pathological conditions [4], [5], [6], [7]. QSAR models as well as other methods are regression, classification or statistical methods used in the chemical and biological sciences, helping in predicting variables or in understanding patterns [8], [9], [10], [11]. Data here reported provide information about a set of HO-1 inhibitors, recovered from the Heme Oxygenase Database (HemeOxDB) together with their pIC_50_ (−logIC_50_) [12]. These latter have been used in building up the first hybrid 2D-QSAR model embracing the all set of known HO-1 inhibitors. The model has also been used to predict the HO-1 pIC_50_ for the Food and Drug Administration approved drugs. These latter predicted HO-1 pIC_50_ data are also here reported.

## 2 Experimental design, materials and methods

### 2.1 Dataset preparation

The dataset consists of 382 HO-1 inhibitors which were randomly split three times and then divided into training (131 compounds), invisible training (131 compounds), calibration (60 compounds) sets for model development and a validation set (60 compounds) for invisible model validation. The three splits and four sets have been randomly generated, and their pIC_50_ minimum, maximum and middle are reported in Table 1.

**Table 1:** Analysis of biological endpoints the HO-1 models (pIC_50_).

**Split**	**Set**	**Min**	**Max**	**Middle**
Split 1	Sub-training	3.69	6.55	5.12
	Calibration	3.41	7.22	5.31
	Test	4	6.62	5.31
	Validation	4	7.22	5.61
				
Split 2	Sub-training	3.41	7.22	5.31
	Calibration	3.69	6.95	5.32
	Test	4	7.22	5.61
	Validation	3.98	6.56	5.27
				
Split 3	Sub-training	3.69	7.22	5.45
	Calibration	3.41	7.22	5.31
	Test	4	6.95	5.47
	Validation	3.78	5.77	4.77

### 2.2 2D-QSAR model development

2D-QSAR models have been developed with the use of the software CORAL [13], [14], [15]. Once the splits and sets were determined, nine models were developed and statistical quality recorded. Differences of these models consist in the way molecular structures have been depicted for the software process. Thus, in Table 2 regressions for the HO-1 pIC_50_ models using SMILES, molecular graphs and hybrid descriptors (SMILES and graph together) are reported. While in Table 3 is reported the statistical quality of models of the HO-1 pIC_50_.

**Table 2:** Regression for the HO-1 pIC_50_ models.

**Model**	**Split**	**Regression equation**
**Hybrid**	Split 1	pIC_50_=0.0000163(±0.0147044)+0.0473151(±0.0001566)*DCW(0,41)
	Split 2	pIC_50_=−0.0264725(±0.0163573)+0.0471089(±0.0001704)*DCW(0,33)
	Split 3	pIC_50_=0.0038812(±0.0212544)+0.0354836(±0.0001711)*DCW(2,30)
		
**SMILES**	Split 1	pIC_50_=1.7303408(±0.0126104)+0.0966933(±0.0004343)*DCW(0,36)
	Split 2	pIC_50_=2.1559955(±0.0115205)+0.0847448(±0.0004112*DCW(0,31)
	Split 3	pIC_50_=2.2807230(±0.0150322)+0.0670793(±0.0004516)*DCW(2,30)
		
**Graph**	Split 1	pIC_50_=0.1566205(±0.0185526)+0.0603249(±0.0002615)*DCW(0,40)
	Split 2	pIC_50_=0.0038015(±0.0250024)+0.0618955(±0.0003421)*DCW(0,34)
	Split 3	pIC_50_=−0.0000202(±0.0224837)+).0724072(±0.0003652)*DCW(2,70)

**Table 3:** Statistical quality for models of HO-1 pIC_50_.

**Model**	**Split**	**Set**	T^*^	N^*^	***n***	***r***^**2**^	***q***^**2**^	***s***	***F***_**calc**_	***F***_(0.05,1,n–2)_	***p*****-value**
**Hybrid**	Split 1	Sub-training	0	41	131	0.8085	0.8033	0.337	545	253.33	0.034
		Calibration			131	0.8029	0.7971	0.390	526	253.33	0.035
		Test			60	0.8183	0.8053	0.381	261	252.12	0.049
		Validation			60	0.8291		0.398	281	252.12	0.047
	Split 2	Sub-training	0	33	131	0.7782	0.7721	0.414	453	253.33	0.037
		Calibration			131	0.8187	0.8130	0.349	582	253.33	0.033
		Test			60	0.8888	0.8801	0.302	464	252.12	0.037
		Validation			60	0.7940		0.515	223	252.12	0.053
	Split 3	Sub-training	2	30	131	0.7263	0.7177	0.427	342	253.33	0.043
		Calibration			131	0.7265	0.7192	0.438	343	253.33	0.043
		Test			60	0.8189	0.8037	0.502	262	252.12	0.049
		Validation			60	0.8204	0.562		265	252.12	0.049
											
**SMILES**	Split 1	Sub-training			131	0.6933	0.6849	0.427	292	253.33	0.047
		Calibration			131	0.6590	0.6497	0.505	249	253.33	0.050
		Test			60	0.5008	0.4714	0.569	58	252.12	0.100
		Validation			60	0.6290		0.551	98	252.12	0.080
	Split 2	Sub-training			131	0.6508	0.6415	0.520	240	253.33	0.051
		Calibration			131	0.6883	0.6790	0.455	285	253.33	0.047
		Test			60	0.7593	0.7400	0.446	183	252.12	0.059
		Validation			60	0.4645		0.688	50	252.12	0.112
	Split 3	Sub-training			131	0.6141	0.6010	0.507	205	253.33	0.057
		Calibration			131	0.6096	0.5994	0.527	201	253.33	0.056
		Test			60	0.6697	0.6431	0.620	118	252.12	0.073
		Validation			60	0.5006		0.614	58	252.12	0.104
											
**Graph**	Split 1	Sub-training	0	40	131	0.7115	0.7035	0.414	318	253.33	0.047
		Calibration			131	0.7077	0.6980	0.470	312	253.33	0.045
		Test			60	0.6839	0.6616	0.483	126	252.12	0.071
		Validation			60	0.6751		0.502	121	252.12	0.072
	Split 2	Sub-training	0	34	131	0.6717	0.6613	0.504	264	253.33	0.049
		Calibration			131	0.7293	0.7209	0.444	348	253.33	0.043
		Test			60	0.7247	0.6941	0.452	153	252.12	0.064
		Validation			60	0.7021		0.543	137	252.12	0.068
	Split 3	Sub-training	2	70	131	0.7336	0.7247	0.421	355	253.33	0.042
		Calibration			131	0.7336	0.7263	0.441	355	253.33	0.042
		Test			60	0.7070	0.6811	0.573	140	252.12	0.067
		Validation			60	0.5712		0.659	77	252.12	0.090

T^*^ and N^*^ are preferable values for the threshold and the number of epochs, respectively; n is the number of compounds in the set; *r*^2^ is the correlation coefficient; *q*^2^ is the cross-validated correlation coefficient; *s* is the root-mean-square error; *F* is the Fisher *F* ratio; *F*_(0.05,1,n–2)_ is the 0.05-quantile of the Fisher's distribution *F*_(1,n–2)_; *p*-value is the Fisher test's significance level.

### 2.3 2D-QSAR model settings for the best model [hybrid model split 1]

**Fig. 1:**
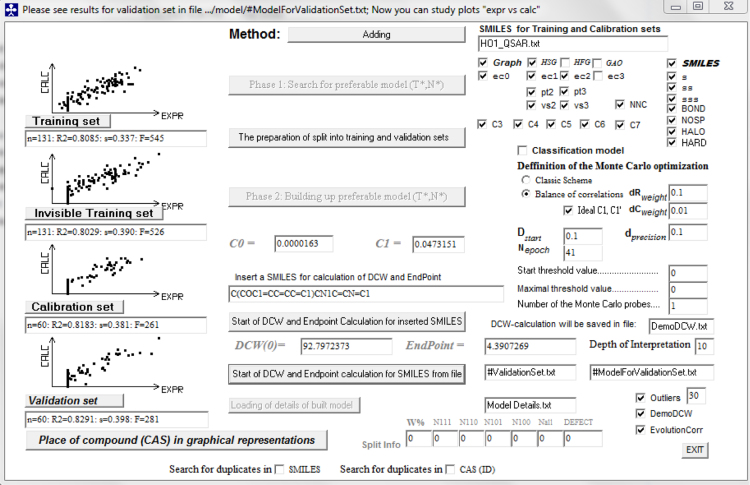
CORAL software validation method for the HO-1 pIC_50_ hybrid model [hybrid model split 1].

Fig. 1 shows a CORAL screenshot with settings for hybrid model split 1. While in Table 4, the complete list of SMILES and their distribution into the sub-training (+), calibration (−), test (#) and validation (*) sets for HO-1 pIC_50_ hybrid model split 1 is reported. These data may be prospectively used in finding novel models for HO-1 inhibition.

**Table 4:** List of SMILES and their distribution into the sub-training (+), calibration (–), test (#) and validation (*) for hybrid model split 1.

	HemeOxDB_ID	SMILES	Exp pIC_50_
+	HemeOxDB131	CSC[C@@H]1CO[C@@](CCC2=CC=C(Cl)C=C2)(CN2C=CN=C2)O1	5.046
+	HemeOxDB306	C(CC1(CN2C=NC(=C2)C2=CC=CC=C2)OCCO1)C1=CC=CC=C1	4
+	HemeOxDB272	COC1=CC=C(CN2C(CN3CCCC3)=NC3=C2C=CC=C3)C=C1	4
+	HemeOxDB247	BrC1=CC=CC(OCCCN2C=NC=N2)=C1	4
+	HemeOxDB55	C[C@@H]1CO[C@@](CCC2=CC=CC=C2)(CN2C=CN=C2)O1	5.699
+	HemeOxDB20	C(CC1(CN2C=CN=C2)OCCO1)C1=CC=CC=C1	6.155
+	HemeOxDB81	O=C(CN1C=NC=N1)C1=CC=C(CC2=CC=CC=C2)C=C1	5.569
+	HemeOxDB34	OC(CN1C=CN=C1)C1=CC=C(Cl)C=C1	5.924
+	HemeOxDB162	[O-][N+](=O)C1=CC=C(C=C1)C(=O)CN1C=NC=N1	4.717
+	HemeOxDB59	O=C(CN1C=CN=C1)C1=CC=C(C=C1)C1=CC=CC=C1	5.678
+	HemeOxDB113	ClC1=CC=C(CC[C@@]2(CN3C=CN=C3)OC[C@@H](CSC3=CC=CC(Br)=C3)O2)C=C1	5.301
+	HemeOxDB185	BrC1=CC=CC(OCCCCCCN2C=CN=C2)=C1	4.469
+	HemeOxDB219	O=C(CCN1C=CN=C1)C1=CC=CC=C1	4.102
+	HemeOxDB107	O=C(CN1C=CN=C1)C1=CC2=C(CCCC2)C=C1	5.398
+	HemeOxDB197	BrC1=CC=CC=C1CN1C(CN2CCCC2)=NC2=C1C=CC=C2	4.377
+	HemeOxDB196	O=C(CCC1=CC=CC=C1)CN1C=NC(=C1C1=CC=CC=C1)C1=CC=CC=C1	4.398
+	HemeOxDB99	O=C(CCC1=CC=CC=C1)CN1C=CN=C1	5.398
+	HemeOxDB91	ClC1=CC=C(CC[C@@]2(CN3C=CN=C3)OC[C@@H](COC3=CC=C(Br)C=C3)O2)C=C1	5.456
+	HemeOxDB258	CCCN1C(CN2CCCC2)=NC2=C1C=CC=C2	4
+	HemeOxDB369	O=C(CCC1=CC=CC=C1)CN1N=NN=C1C1=CC=CC=C1	4
+	HemeOxDB95	ClC1=CC=C(CC[C@@]2(CN3C=CN=C3)OC[C@@H](CN=[N+]=[N-])O2)C=C1	5.444
+	HemeOxDB122	ClC1=CC=C(CC[C@@]2(CN3C=CN=C3)OC[C@@H](CSC3=C(Br)C=CC=C3)O2)C=C1	5.222
+	HemeOxDB373	O=C1C(CC2=C1C=CC=C2)N1C=CN=C1	4
+	HemeOxDB125	OC(CCC1=CC=CC=C1)CN1C=CN=C1	5.208
+	HemeOxDB35	FC[C@@H]1CO[C@@](CCC2=CC=C(Cl)C=C2)(CN2C=CN=C2)O1	5.921
+	HemeOxDB23	O=C(CN1C=NC=N1)C1=CC=C2C=CC=CC2=C1	6.155
+	HemeOxDB261	CSC1=CC=C(CN2C(CN3CCCC3)=NC3=C2C=CC=C3)C=C1	4
+	HemeOxDB80	BrC1=CC=C(C=C1)C(=O)CN1C=NC=N1	5.569
+	HemeOxDB198	IC1=CC=C(OCCCCCCN2C=CN=C2)C=C1	4.377
+	HemeOxDB339	COC1=CC=C(CCN2CCC(CC2)NC2=NC3=CC=CC=C3N2)C=C1	4
+	HemeOxDB143	C[C@H]1CO[C@](CCC2=CC=C(Cl)C=C2)(CN2C=CN=C2)O1	4.921
+	HemeOxDB233	COC(=O)NC1=NC2=C(N1)C=CC(=C2)C(=O)C1=CC=C(F)C=C1	4
+	HemeOxDB178	[H]C1=CC2=C(OC(SCCCCN3C=CN=C3)=N2)C=C1	4.51
+	HemeOxDB280	IC1=CC=C(CN2C(CN3CCCC3)=NC3=C2C=CC=C3)C=C1	4
+	HemeOxDB92	C(CCC1=CC=CC=C1)CN1C=CN=C1	5.456
+	HemeOxDB275	C(C1CCCCC1)N1C(CN2CCCC2)=NC2=C1C=CC=C2	4
+	HemeOxDB377	ON=C(N1C=CN=C1)C(=O)C1=CC=C(Cl)C=C1	3.987
+	HemeOxDB157	O=C(CN1C=NC=N1)C1=CC=C2OCCOC2=C1	4.745
+	HemeOxDB187	ClC1=CC2=C(SC(SCCCCCN3C=CN=C3)=N2)C=C1	4.44
+	HemeOxDB330	CN1C(NCCC2=CNC=N2)=NC2=CC=CC=C12	4
+	HemeOxDB100	ClC1=CC=C(C=C1)C(=O)CN1C=CN=C1	5.398
+	HemeOxDB138	ClC1=CC=C(CSC(CN2C=CN=C2)C2=CC=C(Cl)C=C2Cl)C=C1	4.959
+	HemeOxDB43	NC1=CC=CC(SC[C@@H]2CO[C@](CCC3=CC=C(Cl)C=C3)(CN3C=CN=C3)O2)=C1	5.824
+	HemeOxDB120	ClC1=CC(Cl)=C(COC(CN2C=CN=C2)C2=CC=C(Cl)C=C2Cl)C=C1	5.237
+	HemeOxDB210	ClC1=CC=C(CN2C(CN3CCCC3)=NC3=C2C=CC=C3)C(Cl)=C1	4.208
+	HemeOxDB358	O=C(CCC1=CC=CC=C1)CN1C=CN=C1C1=CC=CC=C1	4
+	HemeOxDB340	CS(=O)(=O)C1=NC=CN1CC(=O)CCC1=CC=CC=C1	4
+	HemeOxDB366	O=C(CCC1=CC=CC=C1)CN1N=CC=N1	4
+	HemeOxDB234	O=C(NCCCN1C=CN=C1)C1=CC=CC=C1	4
+	HemeOxDB50	BrC1=CC(=CC=C1)C(=O)CN1C=NC=N1	5.745
+	HemeOxDB16	ClC1=CC=C(CCCCN2C=CN=C2)C=C1	6.301
+	HemeOxDB379	CCC(O)CN1C=CN=C1	3.883
+	HemeOxDB164	C[C@@H]1CO[C@](CCC2=CC=C(Cl)C=C2)(CN2C=CN=C2)O1	4.699
+	HemeOxDB308	C(CC1(CN2N=CC=N2)OCCO1)C1=CC=CC=C1	4
+	HemeOxDB365	O=C(CCC1=CC=CC=C1)CN1N=C2C=CC=CC2=N1	4
+	HemeOxDB154	CC1=CC=C(C=C1)S(=O)(=O)OC[C@H]1CO[C@](CCC2=CC=C(Cl)C=C2)(CN2C=CN=C2)O1	4.77
+	HemeOxDB278	O=N(=O)C1=CC=CC(CN2C(CN3CCCC3)=NC3=C2C=CC=C3)=C1	4
+	HemeOxDB352	CSC1=NN=NN1CC(=O)CCC1=CC=CC=C1	4
+	HemeOxDB248	IC1=CC=C(OCCCN2C=CN=C2)C=C1	4
+	HemeOxDB189	NC1=CC(SC[C@H]2CO[C@@](CCC3=CC=C(Cl)C=C3)(CN3C=CN=C3)O2)=CC=C1	4.42
+	HemeOxDB245	[O-][N+](=O)C1=CC=C(OCCCN2C=CN=C2)C=C1	4
+	HemeOxDB349	CSC1=NN(CC(=O)CCC2=CC=CC=C2)C=N1	4
+	HemeOxDB361	O=C(CCC1=CC=CC=C1)CN1C=NC(C#N)=C1C#N	4
+	HemeOxDB132	O=C(CCC1=CC=CC=C1)CN1C=NC(=N1)C1=CC=CC=C1	5.046
+	HemeOxDB317	CC1=NC=CN1CC(=O)CCC1=CC=C(Br)C=C1	4
+	HemeOxDB133	O=C(CCC1=CC=CC=C1)CN1N=CN=N1	5.018
+	HemeOxDB282	ClC1=CC(CN2C(CN3CCCC3)=NC3=C2C=CC=C3)=CC(Cl)=C1	4
+	HemeOxDB363	O=C(CCC1=CC=CC=C1)CN1C=NC2=CC=CC=C12	4
+	HemeOxDB274	O=N(=O)C1=CC=C(CN2C(CN3CCCC3)=NC3=C2C=CC=C3)C=C1	4
+	HemeOxDB148	NC1=CC=C(SC[C@H]2CO[C@](CCC3=CC=C(Cl)C=C3)(CN3C=CN=C3)O2)C=C1	4.83
+	HemeOxDB49	OC1=CC=C(OC[C@@H]2CO[C@](CCC3=CC=C(Cl)C=C3)(CN3C=CN=C3)O2)C=C1	5.745
+	HemeOxDB270	BrC1=CC=C(CN2C(CN3CCCC3)=NC3=C2C=CC=C3)C=C1	4
+	HemeOxDB37	ClC1=C(Cl)C=C(C=C1)C(=O)CN1C=CN=C1	5.907
+	HemeOxDB364	O=C(CCC1=CC=CC=C1)CN1N=C(N=C1C1=CC=CC=C1)C1=CC=CC=C1	4
+	HemeOxDB294	COC(=O)NC1=NC2=CC(=CC=C2N1)C(=O)C1=CC=CC=C1	4
+	HemeOxDB320	CCN1CCN(CC1)C1=NC2=CC=CC=C2N1	4
+	HemeOxDB76	O=C(CCC1=CC=CC=C1)CN1C=NN=N1	5.585
+	HemeOxDB295	NCCC1=CN=CN1	4
+	HemeOxDB163	BrC1=CC=CC(OCCCCCN2C=NC=N2)=C1	4.699
+	HemeOxDB304	C(CC1(CN2C=CC=N2)OCCO1)C1=CC=CC=C1	4
+	HemeOxDB61	ClC1=CC=C(CC[C@@]2(CN3C=CN=C3)OC[C@@H](CSC3=CC=C(Br)C=C3)O2)C=C1	5.678
+	HemeOxDB112	C(C1=CC=CC=C1)C1=CC=C(C=C1)C1(CN2C=CN=C2)OCCO1	5.303
+	HemeOxDB205	CC(N1C=CN=C1)C(=O)C1=CC=CC=C1	4.31
+	HemeOxDB38	ClC1=C(Cl)C=C(C=C1)C(=O)CN1C=NC=N1	5.886
+	HemeOxDB380	C(N1C=NC=N1)C1(OCCO1)C1=CC=CC=C1	3.842
+	HemeOxDB17	ClC1=CC=C(CC[C@@]2(CN3C=CN=C3)OC[C@@H](COC3=CC=CC=C3)O2)C=C1	6.229
+	HemeOxDB375	OC(CCC1=CC=CC=C1)CN1C=NC(=C1C1=CC=CC=C1)C1=CC=CC=C1	4
+	HemeOxDB67	C(N1C=CN=C1)C12CC3CC(CC(C3)C1)C2	5.658
+	HemeOxDB172	ClC1=CC=C(CC[C@@]2(CN3C=CN=C3)OC[C@@H](CSC3=CC=NC=C3)O2)C=C1	4.602
+	HemeOxDB72	O=C(CCC1=CC=CC=C1)CN1C=NC=N1	5.602
+	HemeOxDB161	ClC1=CC=C(C=C1)C1(CN2C=CN=C2)OCCO1	4.721
+	HemeOxDB232	[O-][N+](=O)C1=CC=C(OCCCN2C=NC=N2)C=C1	4
+	HemeOxDB286	ClC1=CC2=C(SC(SCCCCN3C=CN=C3)=N2)C=C1	4
+	HemeOxDB86	C(CC12CC3CC(CC(C3)C1)C2)N1C=CN=C1	5.523
+	HemeOxDB62	FC(F)(F)C1=CC=C(SC[C@@H]2CO[C@@](CCC3=CC=C(Cl)C=C3)(CN3C=CN=C3)O2)C=C1	5.678
+	HemeOxDB381	O=C(CCC1=CC=CC=C1)C[N+]1=CC=NC=C1	3.788
+	HemeOxDB186	ClC1=CC=CC=C1C(N1C=CN=C1)(C1=CC=CC=C1)C1=CC=CC=C1	4.456
+	HemeOxDB382	C1CC(CCC1NC1=NC2=CC=CC=C2N1)C1=CN=CN1	3.699
+	HemeOxDB127	FC1=CC=C(COC(CN2C=CN=C2)C2=CC=C(CCC3=CC=CC=C3)C=C2)C=C1	5.155
+	HemeOxDB173	OC1=C(C=CC=C1)C(=O)CCN1C=CN=C1	4.602
+	HemeOxDB31	IC1=CC=C(OCCCCN2C=CN=C2)C=C1	6
+	HemeOxDB289	N1C=CN=C1	4
+	HemeOxDB378	COC(=O)NC1=NC2=C(N1)C=CC(=C2)S(=O)C1=CC=CC=C1	3.939
+	HemeOxDB214	C(CC1=CC=CC=C1)N1C=CN=C1	4.143
+	HemeOxDB203	OC(CCC1=CC=CC=C1)CN1C=CN=N1	4.357
+	HemeOxDB362	O=C(CCC1=CC=CC=C1)CN1C=NC=C1N(=O)=O	4
+	HemeOxDB212	BrC1=CC=CC(OCCCN2C=CN=C2)=C1	4.161
+	HemeOxDB69	ClC1=CC=C(C(=O)CN2C=CN=C2)C(Cl)=C1	5.658
+	HemeOxDB336	COC(=O)SC1=NC=CN1CC(=O)CCC1=CC=CC=C1	4
+	HemeOxDB130	ClC1=CC=C(CC[C@@]2(CN3C=CN=C3)OC[C@@H](COC3=CC=C(I)C=C3)O2)C=C1	5.046
+	HemeOxDB242	C(CCN1C=CN=C1)CN1C=CN=C1	4
+	HemeOxDB58	FC(F)(F)C1=CN=C(SC[C@H]2CO[C@](CCC3=CC=C(Cl)C=C3)(CN3C=CN=C3)O2)C=C1	5.678
+	HemeOxDB284	BrC1=CC=CC(CN2C(CN3CCCC3)=NC3=C2C=CC=C3)=C1	4
+	HemeOxDB190	BrC1=CC=C(C=C1)C1(CN2C=NC=N2)OCCO1	4.42
+	HemeOxDB134	ClC1=CC=C(CC[C@@]2(CN3C=CN=C3)OC[C@@H](CN3C=CN=C3)O2)C=C1	5
+	HemeOxDB44	BrC1=CC=C(C=C1)C1=CC=C(C=C1)C(=O)CN1C=CN=C1	5.824
+	HemeOxDB13	C(CCC1=CC=C(CCCC[N]2=CCN=C2)C=C1)CN1C=CN=C1	6.398
+	HemeOxDB300	BrC1=CC=C(CNCC2=CN=CC=C2)C=C1	4
+	HemeOxDB119	NC1=CC=C(SC[C@@H]2CO[C@](CCC3=CC=C(Cl)C=C3)(CN3C=CN=C3)O2)C=C1	5.237
+	HemeOxDB9	FC1=CC=C(OC[C@@H]2CO[C@@](CCC3=CC=C(Cl)C=C3)(CN3C=CN=C3)O2)C=C1	6.553
+	HemeOxDB327	ClC1=CC=C(C=C1)C(/CN1C=CN=C1)=N/NC1=CC=CC=C1	4
+	HemeOxDB353	FC(F)(F)C(=O)N1C=CN=C1	4
+	HemeOxDB273	FC(F)(F)C1=CC=C(CN2C(CN3CCCC3)=NC3=C2C=CC=C3)C=C1	4
+	HemeOxDB26	CC1=CC=C(C=C1)S(=O)(=O)C[C@H]1CO[C@@](CCC2=CC=C(Cl)C=C2)(CN2C=CN=C2)O1	6.097
+	HemeOxDB97	O=S(CCCN1C=CN=C1)C1=CC=CC=C1	5.432
+	HemeOxDB311	C1C2CC3CC1CC(C2)C3N1C=CN=C1	4
+	HemeOxDB165	ClC1=CC=C(CCC2(CN3C=CN=C3)OCCCO2)C=C1	4.699
+	HemeOxDB305	C(CC1(CN2C=CN=N2)OCCO1)C1=CC=CC=C1	4
+	HemeOxDB228	C(CCCN1C=CN=C1)CCN1C=CN=C1	4
+	HemeOxDB180	BrC1=CC=C(OCCCCN2C=CN=C2)C=C1	4.509
+	HemeOxDB204	O/N=C(/CN1C=CN=C1)C1=CC=C(Cl)C=C1	4.337
–	HemeOxDB303	BrC1=NN(CC(=O)CCC2=CC=CC=C2)C(Br)=N1	4
–	HemeOxDB267	ClC1=CC=CC=C1CN1C(CN2CCCC2)=NC2=C1C=CC=C2	4
–	HemeOxDB60	BrC1=CC=CC(OCCCCN2C=CN=C2)=C1	5.678
–	HemeOxDB201	C(N1C=CN=C1)C1=CC=CC=C1	4.357
–	HemeOxDB259	C(N1CCCC1)C1=NC2=C(C=CC=C2)N1C(C1=CC=CC=C1)C1=CC=CC=C1	4
–	HemeOxDB137	OC(CCC1=CC=CC=C1)CN1C=NC=N1	4.991
–	HemeOxDB254	COC1=CC=C(CCN2CCC(CC2)NC2=NC3=C(C=CC=C3)N2CC2=CC=C(F)C=C2)C=C1	4
–	HemeOxDB68	ClC1=CC=C(C=C1)C(=O)CN1C=NC=N1	5.658
–	HemeOxDB227	ClC1=CC=C(CC[C@]2(CN3C=CN=C3)OC[C@@H](COC3=CC=C(C=C3)C34CC5CC(CC(C5)C3)C4)O2)C=C1	4
–	HemeOxDB372	O=C(NCCCN1C=CN=C1)NC1=CC=CC=C1	4
–	HemeOxDB90	ClC[C@@H]1CO[C@@](CCC2=CC=C(Cl)C=C2)(CN2C=CN=C2)O1	5.456
–	HemeOxDB11	NC1=CC=C(SC[C@@H]2CO[C@@](CCC3=CC=C(Cl)C=C3)(CN3C=CN=C3)O2)C=C1	6.481
–	HemeOxDB194	NC1=CC=C(SC[C@H]2CO[C@@](CCC3=CC=C(Cl)C=C3)(CN3C=CN=C3)O2)C=C1	4.398
–	HemeOxDB268	O=N(=O)C1=CC=CC=C1CN1C(CN2CCCC2)=NC2=C1C=CC=C2	4
–	HemeOxDB281	C(CN1C(CN2CCCC2)=NC2=C1C=CC=C2)OC1=CC=CC=C1	4
–	HemeOxDB337	COC1=CC=C(CCN2CCC(CC2)NC2=NC3=C(C=C(C)C(C)=C3)N2CC2=CC=C(F)C=C2)C=C1	4
–	HemeOxDB310	C(CCNC1=NC2=CC=CC=C2N1)CCN1CCCCC1	4
–	HemeOxDB238	NC1=C(SC[C@H]2CO[C@](CCC3=CC=C(Cl)C=C3)(CN3C=CN=C3)O2)C=CC=C1	4
–	HemeOxDB88	BrC1=CC=C(C=C1)C(=O)CN1C=CN=C1	5.495
–	HemeOxDB223	C(CCN1C=NC=N1)COC1=CC=CC=C1	4.036
–	HemeOxDB199	BrCC(=O)CCC1=CC=C(Br)C=C1	4.377
–	HemeOxDB64	ClC1=C(Cl)C(Cl)=C(C=C1)C(=O)CN1C=CN=C1	5.677
–	HemeOxDB206	BrC1=C(OCCCCN2C=CN=C2)C=CC=C1	4.276
–	HemeOxDB156	O=C1OC2=C(C=CC=C2)N1CCCCN1C=CN=C1	4.752
–	HemeOxDB331	COC(=O)C1=C(N(CC(=O)CCC2=CC=CC=C2)C=N1)C(=O)OC	4
–	HemeOxDB77	C[C@H]1CO[C@@](CCC2=CC=C(Cl)C=C2)(CN2C=CN=C2)O1	5.585
–	HemeOxDB213	[H][C@@]12CCCC[C@]1([H])OC(CCC1=CC=C(Cl)C=C1)(CN1C=CN=C1)O2	4.161
–	HemeOxDB251	C1=CN(C=N1)C1=CC=CC=C1	4
–	HemeOxDB220	OC(CN1C=NC=N1)(CN1C=NC=N1)C1=C(F)C=C(F)C=C1	4.097
–	HemeOxDB65	FC1=CC=C(SC[C@@H]2CO[C@@](CCC3=CC=C(Cl)C=C3)(CN3C=CN=C3)O2)C=C1	5.658
–	HemeOxDB175	COC1=CC=C(OC[C@H]2CO[C@@](CCC3=CC=C(Cl)C=C3)(CN3C=CN=C3)O2)C=C1	4.553
–	HemeOxDB341	CS(=O)(=O)C1=NC=NN1CC(=O)CCC1=CC=CC=C1	4
–	HemeOxDB383	OC1=CC=C(CCC(=O)CN2C=CN=C2)C=C1	3.418
–	HemeOxDB357	O=C(CCC1=CC=CC=C1)CN1C=CC=N1	4
–	HemeOxDB169	BrC1=CN(CCC(=O)CCC2=CC=CC=C2)C=N1	4.658
–	HemeOxDB195	CC(O)C(CC1=CC=C(Cl)C=C1)N1C=CN=C1	4.398
–	HemeOxDB21	COC1=CC=C(CCC(O)CN2C=CN=C2)C=C1	6.155
–	HemeOxDB14	CC(C)N1CCN(CC1)C1=CC=C(OC[C@@H]2CO[C@](CN3C=NC=N3)(O2)C2=CC=C(Cl)C=C2Cl)C=C1	6.387
–	HemeOxDB338	COC1=CC=C(CCN2CCC(CC2)NC2=NC3=CC(Cl)=C(Cl)C=C3N2CC2=CC=C(F)C=C2)C=C1	4
–	HemeOxDB46	OC1=CC=C(SC[C@@H]2CO[C@@](CCC3=CC=C(Cl)C=C3)(CN3C=CN=C3)O2)C=C1	5.799
–	HemeOxDB155	CC1=CC=C(C=C1)C(=O)CN1C=CN=C1	4.77
–	HemeOxDB56	OC(CCN1C=CN=C1)C12CC3CC(CC(C3)C1)C2	5.699
–	HemeOxDB1	OC(CCC1=CC=C(I)C=C1)CN1C=CN=C1	7.222
–	HemeOxDB158	ClC1=CC2=C(SC(SCCCN3C=CN=C3)=N2)C=C1	4.735
–	HemeOxDB351	CSC1=NN(CCC(=O)CCC2=CC=CC=C2)C=N1	4
–	HemeOxDB321	CCOC(=O)C1=NC=CN1CC(=O)CCC1=CC=CC=C1	4
–	HemeOxDB250	CSCCC(N)C(O)=O	4
–	HemeOxDB41	NC1=CC=C(OC[C@@H]2CO[C@](CCC3=CC=C(Cl)C=C3)(CN3C=CN=C3)O2)C=C1	5.854
–	HemeOxDB174	O=C(CN1C=CN=C1)C1=CC=CC=C1	4.553
–	HemeOxDB293	FC1=CC=C(CN2C(NC3CCNCC3)=NC3=C2C=CC=C3)C=C1	4
–	HemeOxDB177	ClC1=CC(Cl)=C(C=C1)C1(CN2C=CN=C2)OCCO1	4.538
–	HemeOxDB83	ClC1=CC=C(CC[C@@]2(CN3C=CN=C3)OC[C@@H](CSC3=CC=C(Cl)C=C3)O2)C=C1	5.553
–	HemeOxDB239	NC(CC1=CN=CN1)C(O)=O	4
–	HemeOxDB257	N#CC1=CC=C(CN2C(CN3CCCC3)=NC3=C2C=CC=C3)C=C1	4
–	HemeOxDB360	O=C(CCC1=CC=CC=C1)CN1C=NC(=N1)N(=O)=O	4
–	HemeOxDB128	ClC1=C(Cl)C=C(C=C1)C1(CN2C=CN=C2)OCCO1	5.097
–	HemeOxDB144	BrC1=CC=C(C=C1)C1(CN2C=CN=C2)OCCO1	4.921
–	HemeOxDB54	ClC1=CC=C(CC[C@@]2(CN3C=CN=C3)OC[C@@H](COC3=CC=C(C=C3)C3=CC=CC=C3)O2)C=C1	5.699
–	HemeOxDB253	CC1=NC=CN1	4
–	HemeOxDB265	CC1=CC=CC(CN2C(CN3CCCC3)=NC3=C2C=CC=C3)=C1	4
–	HemeOxDB335	COC(=O)NC1=NC2=CC(=CC=C2N1)C(=O)C1=CC=CS1	4
–	HemeOxDB87	ClC1=CC=C(CC[C@@]2(CN3C=CN=C3)OC[C@@H](CSC#N)O2)C=C1	5.523
–	HemeOxDB347	CSC1=NC=CN1CCC(=O)CCC1=CC=CC=C1	4
–	HemeOxDB183	ClC1=CC=C(C=C1)C(=O)CCN1C=CN=C1	4.495
–	HemeOxDB117	NC1=CC=CC(SC[C@H]2CO[C@](CCC3=CC=C(Cl)C=C3)(CN3C=CN=C3)O2)=C1	5.284
–	HemeOxDB191	COC1=CC=C(C=C1)C(=O)CN1C=CN=C1	4.409
–	HemeOxDB208	OC(CCC1=CC=CC=C1)CN1C=NN=N1	4.252
–	HemeOxDB298	BrC1=CC=C(C=C1)C(=O)CN=[N+]=[N-]	4
–	HemeOxDB332	COC(=O)C1=CN(CC(=O)CCC2=CC=CC=C2)C=N1	4
–	HemeOxDB376	OC(CCC1=CC=CC=C1)CN1N=CN=N1	4
–	HemeOxDB230	BrC1=CC=CC(OCCCCN2C=NC=N2)=C1	4
–	HemeOxDB101	O=C(CN1C=CN=C1)C1=CC=C(CCC2=CC=CC=C2)C=C1	5.398
–	HemeOxDB116	FC1=CC=C(COC(CN2C=CN=C2)C2=CC=C(C=C2)C2=CC=C(Br)C=C2)C=C1	5.301
–	HemeOxDB290	CN1C=CN=C1	4
–	HemeOxDB216	O=C(CN1C=CN=C1)C1=CC=C2OCCOC2=C1	4.137
–	HemeOxDB51	BrC1=CC=C(CCC2(CN3C=CN=C3)OCCO2)C=C1	5.721
–	HemeOxDB12	BrC1=CC=C(CCC(=O)CN2C=NC=N2)C=C1	6.409
–	HemeOxDB4	OC(CCC1=CC=C(Br)C=C1)CN1C=CN=C1	6.854
–	HemeOxDB279	N#CC1=CC=CC=C1CN1C(CN2CCCC2)=NC2=C1C=CC=C2	4
–	HemeOxDB150	ClC1=CC=C(COC(CN2C=CN=C2)C2=CC=C(Cl)C=C2Cl)C=C1	4.796
–	HemeOxDB345	CS(=O)(=O)C1=NN=NN1CCC(=O)CCC1=CC=CC=C1	4
–	HemeOxDB297	[O-][N+](=O)C1=NC=CN1CC(=O)CCC1=CC=CC=C1	4
–	HemeOxDB10	CC(=O)N1CCN(CC1)C1=CC=C(OC[C@@H]2CO[C@](CN3C=CN=C3)(O2)C2=CC=C(Cl)C=C2Cl)C=C1	6.523
–	HemeOxDB75	O=C(CCN1C=NC=N1)CCC1=CC=CC=C1	5.602
–	HemeOxDB147	ClC1=CC=C(CC[C@]2(CN3C=CN=C3)OC[C@@H](CSC3=CC4=C(C=CC=C4)C=C3)O2)C=C1	4.854
–	HemeOxDB79	FC1=CC=C(CCC(=O)CN2C=CN=C2)C=C1	5.569
–	HemeOxDB33	ClC1=CC=C(CC[C@@]2(CN3C=CN=C3)OC[C@@H](CSC3=CC=CC=C3)O2)C=C1	5.987
–	HemeOxDB328	ClC1=CC=C(C=C1)C(=O)CN1C=NN=C1	4
–	HemeOxDB359	O=C(CCC1=CC=CC=C1)CN1C=NC(=C1)N(=O)=O	4
–	HemeOxDB114	O=C(CN1C=CN=C1)C1=CC=C(C=C1)C1CCCCC1	5.301
–	HemeOxDB342	CS(=O)(=O)C1=NC=NN1CCC(=O)CCC1=CC=CC=C1	4
–	HemeOxDB73	O=C(CN1C=CN=C1)C1=CC=C(C=C1)N(=O)=O	5.602
–	HemeOxDB367	O=C(CCC1=CC=CC=C1)CN1N=NC(=N1)C1=CC=CC=C1	4
–	HemeOxDB19	ClC1=CC=C(CC[C@]2(CN3C=CN=C3)OC[C@@H](COC3=CC=C(C=C3)C#N)O2)C=C1	6.174
–	HemeOxDB302	BrC1=CN=CN1CCC(=O)CCC1=CC=CC=C1	4
–	HemeOxDB63	FC1=CC=C(C=C1)C(=O)CN1C=CN=C1	5.678
–	HemeOxDB123	CC1=CC=C(C=C1)S(=O)(=O)OC[C@@H]1CO[C@@](CCC2=CC=C(Cl)C=C2)(CN2C=CN=C2)O1	5.222
–	HemeOxDB141	OC[C@@H]1CO[C@@](CCC2=CC=C(Cl)C=C2)(CN2C=CN=C2)O1	4.921
–	HemeOxDB277	ClC1=C(Cl)C=C(CN2C(CN3CCCC3)=NC3=C2C=CC=C3)C=C1	4
–	HemeOxDB229	CC(=O)NCCC1=CNC=N1	4
–	HemeOxDB102	NC1=CC=CC(SC[C@@H]2CO[C@@](CCC3=CC=C(Cl)C=C3)(CN3C=CN=C3)O2)=C1	5.398
–	HemeOxDB264	C(N1CCCC1)C1=NC2=C(C=CC=C2)N1CC1=CC2=C(C=CC=C2)C=C1	4
–	HemeOxDB237	CN(C)CCC(=O)C1=CC=C(Cl)C=C1	4
–	HemeOxDB354	FC1=CC=C(C=C1)C1=CC=NO1	4
–	HemeOxDB159	ClC1=CC(C(=O)CN2C=NC=N2)=C(Cl)C=C1	4.735
–	HemeOxDB256	C(N1CCCC1)C1=NC2=C(N1)C=CC=C2	4
–	HemeOxDB241	CC(C)C1=NC=CN1CC(=O)C1=CC=C(Br)C=C1	4
–	HemeOxDB368	O=C(CCC1=CC=CC=C1)CN1N=NC2=C1C=CC=C2	4
–	HemeOxDB333	COC(=O)C1=NC=NN1CC(=O)CCC1=CC=CC=C1	4
–	HemeOxDB146	C(CN1C=CN=C1)CC1=CC=CC=C1	4.854
–	HemeOxDB78	C(N1C=CN=C1)C1(OCCO1)C1=CC2=CC=CC=C2C=C1	5.58
–	HemeOxDB57	BrC1=CC=CC(=C1)C(=O)CN1C=CN=C1	5.686
–	HemeOxDB312	C1CC(CCN1)NC1=NC2=C(N1)C=CC=C2	4
–	HemeOxDB32	[H]C1=CC2=C(SC(SCCCCN3C=CN=C3)=N2)C=C1	6
–	HemeOxDB324	CCOC(=O)N1CCC(CC1)NC1=NC2=C(C=CC=C2)N1CC1=CC=C(F)C=C1	4
–	HemeOxDB193	[H]C1=CC2=C(SC(SCCCCCN3C=CN=C3)=N2)C=C1	4.4
–	HemeOxDB103	C(CCN1C=CN=C1)COC1=CC=CC=C1	5.398
–	HemeOxDB291	COC1=C2C(=O)[C@]3(OC2=C(Cl)C(OC)=C1)[C@H](C)CC(=O)C=C3OC	4
–	HemeOxDB283	ClC1=CC=CC(Cl)=C1CN1C(CN2CCCC2)=NC2=C1C=CC=C2	4
–	HemeOxDB222	O=C(CCC1=CC=CC=C1)CN1C=CN=N1	4.051
–	HemeOxDB110	ClC1=CC=C(CCC2(CN3C=CN=C3)SCCS2)C=C1	5.328
–	HemeOxDB355	NC1=NC2=C(N1)C=CC(OCCCCN1CCCCC1)=C2	4
–	HemeOxDB288	NC1=C(C=CC(Cl)=C1)C1=NN=NN1	4
–	HemeOxDB28	ClC1=CC=C(CC[C@@]2(CN3C=CN=C3)OC[C@@H](CSC3=CC4=C(C=CC=C4)C=C3)O2)C=C1	6.046
–	HemeOxDB7	BrC1=CC=C(CCCCN2C=CN=C2)C=C1	6.602
–	HemeOxDB329	ClC1=CC=C(OCCCCCNC2=NC3=CC=CC=C3N2)C=C1	4
–	HemeOxDB129	ClC1=CC=C(C=C1)C(/CN1C=CN=C1)=N/OCC1=CC=C(Br)C=C1	5.081
–	HemeOxDB160	O=S(CCCCN1C=CN=C1)C1=CC=CC=C1	4.734
–	HemeOxDB27	[H]C1=CC2=C(SC(SCCCN3C=CN=C3)=N2)C=C1	6.046
–	HemeOxDB5	OC(CN1C=CN=C1)C1=CC=C(CCC2=CC=CC=C2)C=C1	6.648
–	HemeOxDB225	CCCOC1=CC=C2NC(NC(=O)OC)=NC2=C1	4
#	HemeOxDB142	O=C(CN1C=CN=C1)NCC1=CC=CC=C1	4.921
#	HemeOxDB66	O=C(CN1C=CN=C1)C1=CC=CC2=CC=CC=C12	5.658
#	HemeOxDB96	IC1=CC=C(CCC2(CN3C=CN=C3)OCCO2)C=C1	5.432
#	HemeOxDB167	CC1=CC=C(C=C1)S(=O)(=O)OC[C@H]1CO[C@@](CCC2=CC=C(Cl)C=C2)(CN2C=CN=C2)O1	4.678
#	HemeOxDB39	COC1=CC=C(OC[C@@H]2CO[C@](CCC3=CC=C(Cl)C=C3)(CN3C=CN=C3)O2)C=C1	5.876
#	HemeOxDB89	ClC1=CC=C(C=C1)C(CN1C=CN=C1)NCC1=CC=CC=C1	5.47
#	HemeOxDB252	COC1=CC=CC(=C1)C1=NN=NN1	4
#	HemeOxDB151	C(N1C=CN=C1)C1(OCCO1)C1=CC=C(C=C1)C1=CC=CC=C1	4.79
#	HemeOxDB71	C(CSC1=CC=CC=C1)CN1C=CN=C1	5.62
#	HemeOxDB84	C(CCN1C=CN=C1)CCC1=CC=CC=C1	5.553
#	HemeOxDB6	O=C(CN1C=NC=N1)C1=CC2=C(CCCC2)C=C1	6.62
#	HemeOxDB226	ClC1=CC=C(CC[C@@]2(CN3C=CN=C3)OC[C@@H](COC3=CC=C(C=C3)C34CC5CC(CC(C5)C3)C4)O2)C=C1	4
#	HemeOxDB40	OC(CCC1=CC=C(F)C=C1)CN1C=CN=C1	5.854
#	HemeOxDB109	O=C(CN1C=CN=C1)C1=CC2=C(CCC2)C=C1	5.337
#	HemeOxDB299	BrC1=CC=C(CCC(=O)CN2C=NC3=CC=CC=C23)C=C1	4
#	HemeOxDB249	C(OC1=CC=C(CN2C(CN3CCCC3)=NC3=C2C=CC=C3)C=C1)C1=CC=CC=C1	4
#	HemeOxDB350	CSC1=NN(CC(=O)CCC2=CC=CC=C2)N=N1	4
#	HemeOxDB149	OC(CCC1=CC=CC=C1)CN1C=NC(=C1)C1=CC=CC=C1	4.824
#	HemeOxDB104	BrC1=CC(=CC=C1)C1(CN2C=CN=C2)OCCO1	5.398
#	HemeOxDB246	[O-][N+](=O)C1=CC=C(OCCCCN2C=NC=N2)C=C1	4
#	HemeOxDB326	ClC1=C(Cl)N(CC(=O)CCC2=CC=CC=C2)C=N1	4
#	HemeOxDB356	O=C(C1CC1)C1=CC=C(C=C1)N1C=CN=C1	4
#	HemeOxDB325	CCOC(=O)N1CCC(CC1)NC1=NC2=C(N1)C=CC=C2	4
#	HemeOxDB217	IC1=CC=C(OCCCN2C=NC=N2)C=C1	4.125
#	HemeOxDB53	O=C(CN1C=CN=C1)C1=CC=C(CC2=CC=CC=C2)C=C1	5.701
#	HemeOxDB276	ClC1=C(Cl)C(CN2C(CN3CCCC3)=NC3=C2C=CC=C3)=CC=C1	4
#	HemeOxDB29	FC1=CC=C(COC(CCC2=CC=C(Cl)C=C2)CN2C=CN=C2)C=C1	6.046
#	HemeOxDB136	BrC1=CC=C(OCCCCCCN2C=CN=C2)C=C1	5
#	HemeOxDB313	CC(=O)C(CC1=CC=C(Cl)C=C1)N1C=CN=C1	4
#	HemeOxDB25	O=C(CN1C=NC=N1)C1=CC=CC2=CC=CC=C12	6.102
#	HemeOxDB243	CCC(COC(C)=O)N1C=CN=C1	4
#	HemeOxDB184	O=C(CCC1=CC=CC=C1)CN1C=NC(=C1)C1=CC=CC=C1	4.495
#	HemeOxDB318	CC1=NC=CN1CC(=O)CCC1=CC=CC=C1	4
#	HemeOxDB322	CCOC(=O)CC1=NN(CC(=O)CCC2=CC=CC=C2)N=N1	4
#	HemeOxDB145	C(CC1(CN2C=NC=N2)OCCO1)C1=CC=CC=C1	4.886
#	HemeOxDB346	CSC1=NC=CN1CC(=O)CCC1=CC=CC=C1	4
#	HemeOxDB319	CCCC(=O)N1C=CN=C1	4
#	HemeOxDB244	C(COC1=CC=CC=C1)CN1C=NC=N1	4
#	HemeOxDB74	NC1=C(SC[C@@H]2CO[C@@](CCC3=CC=C(Cl)C=C3)(CN3C=CN=C3)O2)C=CC=C1	5.602
#	HemeOxDB153	[H]C1=CC2=C(OC(SCCCN3C=CN=C3)=N2)C=C1	4.772
#	HemeOxDB108	ClC1=CC(Cl)=C(C=C1)C(=O)CN1C=NC=N1	5.387
#	HemeOxDB236	C(N1CCCC1)C1=NC2=C(C=CC=C2)N1CC1=CC=CC=C1	4
#	HemeOxDB85	O=C(CN1C=CN=C1)C12CC3CC(CC(C3)C1)C2	5.523
#	HemeOxDB36	C(CCN1C=CN=C1)CSC1=CC=CC=C1	5.921
#	HemeOxDB105	C(CN1C=CN=C1)SCC1=CC=CC=C1	5.398
#	HemeOxDB211	ClC1=CC=C(C=C1)C1(CN2C=NC=N2)OCCO1	4.163
#	HemeOxDB70	COC1=CC=C(CCC(=O)CN2C=CN=C2)C=C1	5.658
#	HemeOxDB370	O=C(CCN1C=CC=N1)CCC1=CC=CC=C1	4
#	HemeOxDB202	IC1=CC=C(OCCCCCN2C=CN=C2)C=C1	4.357
#	HemeOxDB181	C(N1C=CN=C1)C1(OCCO1)C1=CC=CC=C1	4.509
#	HemeOxDB139	O=C(CN1C=CN=C1)C1=CC=C(OCC2=CC=CC=C2)C=C1	4.959
#	HemeOxDB314	CC(=O)C(CC1=CC=CC=C1)N1C=NC=N1	4
#	HemeOxDB323	CCOC(=O)CC1=NN=NN1CC(=O)CCC1=CC=CC=C1	4
#	HemeOxDB135	OC(CN1C=CN=C1)C1=CC=C(C=C1)N(=O)=O	5
#	HemeOxDB126	ClC1=CC=C(Cl)C(=C1)C(=O)CN1C=CN=C1	5.18
#	HemeOxDB235	CCCSC1=CC2=C(NC(NC(=O)OC)=N2)C=C1	4
#	HemeOxDB24	O=C(CN1C=NC=N1)C1=CC=C(C=C1)C1=CC=CC=C1	6.131
#	HemeOxDB18	C[C@@H]1CO[C@@](CCC2=CC=C(Cl)C=C2)(CN2C=CN=C2)O1	6.222
#	HemeOxDB82	ClC1=CC=C(CCC2(CN3C=CN=C3)OCCO2)C=C1	5.553
#	HemeOxDB292	COC1=CC=C(CCN2CCC(CC2)NC2=NC3=CC=CC=C3N2CC2=CC=CC=C2)C=C1	4
*	HemeOxDB348	CSC1=NC=NN1CC(=O)CCC1=CC=CC=C1	4
*	HemeOxDB287	COC(=O)NC1=NC2=C(N1)C=CC(SC1=CC=CC=C1)=C2	4
*	HemeOxDB271	CC1=CC=C(CN2C(CN3CCCC3)=NC3=C2C=CC=C3)C=C1	4
*	HemeOxDB118	ClC1=CC=C(C(CN2C=CN=C2)OCC2=C(Cl)C=CC=C2Cl)C(Cl)=C1	5.252
*	HemeOxDB98	FC1=CC=C(CCC2(CN3C=CN=C3)OCCO2)C=C1	5.42
*	HemeOxDB266	N#CC1=CC=CC(CN2C(CN3CCCC3)=NC3=C2C=CC=C3)=C1	4
*	HemeOxDB106	IC1=CC=C(C=C1)C(=O)CN1C=CN=C1	5.398
*	HemeOxDB121	ClC1=CC=C(CC[C@@]2(CN3C=CN=C3)OC[C@@H](CSC3=CC=C(C=C3)N(=O)=O)O2)C=C1	5.222
*	HemeOxDB115	NC1=CC=CC=C1SC[C@@H]1CO[C@](CCC2=CC=C(Cl)C=C2)(CN2C=CN=C2)O1	5.301
*	HemeOxDB296	CCCCN1C=CN=C1	4
*	HemeOxDB170	O=C(CCN1C=CN=C1)/C=C/C1=CC=CC=C1	4.638
*	HemeOxDB371	O=C(CN1C=CN=C1)C1=CC=CS1	4
*	HemeOxDB111	ClC1=CC=C(CCC(=O)CN2C=CN=C2)C=C1	5.328
*	HemeOxDB45	O=C(CCCC1=CC=CC=C1)CN1C=CN=C1	5.824
*	HemeOxDB168	NC[C@H]1CO[C@@](CCC2=CC=CC=C2)(CN2C=CN=C2)O1	4.678
*	HemeOxDB192	C(CC1(CN2C=NN=N2)OCCO1)C1=CC=CC=C1	4.409
*	HemeOxDB93	O=C(CCN1C=CN=C1)C12CC3CC(CC(C3)C1)C2	5.456
*	HemeOxDB188	IC1=CC=C(OCCCCN2C=NC=N2)C=C1	4.42
*	HemeOxDB224	ClC1=CC=C(Cl)C(CN2C(CN3CCCC3)=NC3=C2C=CC=C3)=C1	4.018
*	HemeOxDB215	C(CC1(CN2N=CN=N2)OCCO1)C1=CC=CC=C1	4.143
*	HemeOxDB231	ClC1=CC=C(CN2C(CN3CCCC3)=NC3=C2C=CC=C3)C=C1	4
*	HemeOxDB171	O=C(C(N1C=CN=C1)C1=CC=CC=C1)C1=CC=CC=C1	4.62
*	HemeOxDB334	COC(=O)C1=NN(CC(=O)CCC2=CC=CC=C2)C=N1	4
*	HemeOxDB307	C(CC1(CN2C=NC(=C2C2=CC=CC=C2)C2=CC=CC=C2)OCCO1)C1=CC=CC=C1	4
*	HemeOxDB182	C(CN1C=CN=C1)OCC1=CC=CC=C1	4.495
*	HemeOxDB124	C(CN1C=CN=C1)SC1=CC=CC=C1	5.222
*	HemeOxDB140	O=C(CN1C=NC=N1)C1=CC=CC=C1	4.924
*	HemeOxDB343	CS(=O)(=O)C1=NN(CC(=O)CCC2=CC=CC=C2)C=N1	4
*	HemeOxDB221	NC1=C(SC[C@H]2CO[C@@](CCC3=CC=C(Cl)C=C3)(CN3C=CN=C3)O2)C=CC=C1	4.06
*	HemeOxDB316	CC(C)(N1C=CN=C1)C(=O)C1=CC=CC=C1	4
*	HemeOxDB374	O=C1C(CC2=CC=CC=C12)N1C=CN=C1	4
*	HemeOxDB240	NCC(=O)C1=CC=C(Br)C=C1	4
*	HemeOxDB269	ClC1=CC=CC(CN2C(CN3CCCC3)=NC3=C2C=CC=C3)=C1	4
*	HemeOxDB209	C(CN1C=CN=C1)OC1=CC=CC=C1	4.215
*	HemeOxDB52	O=C(CN1C=CN=C1)C1=CC=C2C=CC=CC2=C1	5.721
*	HemeOxDB48	COC[C@@H]1CO[C@@](CCC2=CC=C(Cl)C=C2)(CN2C=CN=C2)O1	5.762
*	HemeOxDB218	CC1=CC=CC=C1CN1C(CN2CCCC2)=NC2=C1C=CC=C2	4.119
*	HemeOxDB22	COC1=CC=C(SC[C@@H]2CO[C@@](CCC3=CC=C(Cl)C=C3)(CN3C=CN=C3)O2)C=C1	6.155
*	HemeOxDB262	FC1=CC=C(CN2C(CN3CCCC3)=NC3=C2C=CC=C3)C=C1	4
*	HemeOxDB2	OC(CN1C=CN=C1)C1=CC=C(C=C1)C1=CC=C(Br)C=C1	7.222
*	HemeOxDB3	IC1=CC=C(CCC(=O)CN2C=CN=C2)C=C1	6.959
*	HemeOxDB8	O=C(CC(C1=CC=CC=C1)C1=CC=CC=C1)CN1C=CN=C1	6.569
*	HemeOxDB94	C(N1C=NC=N1)C1(OCCO1)C1=CC2=CC=CC=C2C=C1	5.446
*	HemeOxDB47	BrC1=CC=C(CCC(=O)CN2C=CN=C2)C=C1	5.77
*	HemeOxDB166	NC[C@H]1CO[C@](CCC2=CC=C(Cl)C=C2)(CN2C=CN=C2)O1	4.678
*	HemeOxDB263	C(CC1=CC=CC=C1)N1C(CN2CCCC2)=NC2=C1C=CC=C2	4
*	HemeOxDB152	O=C1SC2=C(C=CC=C2)N1CCCN1C=CN=C1	4.78
*	HemeOxDB301	BrC1=CN(CC(=O)CCC2=CC=CC=C2)C=N1	4
*	HemeOxDB285	N1C=NC2=CC=CC=C12	4
*	HemeOxDB309	C(CC1=CC=CS1)N1C=CN=C1	4
*	HemeOxDB315	CC(=O)CCC1=CC=C(Br)C=C1	4
*	HemeOxDB30	ClC1=CC=C(CC[C@@]2(CN3C=CN=C3)OC[C@@H](CSC3CCCCC3)O2)C=C1	6.027
*	HemeOxDB200	BrC1=CC=CC(OCCCCCN2C=CN=C2)=C1	4.371
*	HemeOxDB15	OC(CCC1=CC=C(Cl)C=C1)CN1C=CN=C1	6.301
*	HemeOxDB176	O=C(CCN1C=CN=C1)CCC1=CC=CC=C1	4.553
*	HemeOxDB344	CS(=O)(=O)C1=NN(CCC(=O)CCC2=CC=CC=C2)N=N1	4
*	HemeOxDB207	COC(=O)C1=CN=CN1CC(=O)CCC1=CC=CC=C1	4.26
*	HemeOxDB255	OC(=O)CN1C=CN=C1	4
*	HemeOxDB260	CC(C)C1=CC=C(CN2C(CN3CCCC3)=NC3=C2C=CC=C3)C=C1	4
*	HemeOxDB179	C(COC1=CC=CC=C1)CN1C=CN=C1	4.509

### 2.4 QSAR hybrid model split 1 validation

The endpoints of the FDA-approved drugs were determined in order to additionally validate the model. The whole set composed of 1428 drugs was refined in order to remove quaternary ammonium salts, and compounds with too long SMILES (not elaborated by CORAL), and compounds containing atoms not enumerated in the model (Al, Fe, Gd, etc.). Overall, the whole set was reduced to 1376 compounds and these were evaluated with hybrid model resulting from split 1. Over 1376 compounds, 995 have been defined as outliers by the model since they fall outside the domain of applicability. Table 5 reports the SMILES and predicted HO-1 pIC_50_ for these FDA approved drugs evaluated with the hybrid model split 1.

**Table 5:** List of SMILES and predicted pIC_50_ of the FDA-approved drugs.

	Calc pIC_50_
CCC1=C(C)CN(C(=O)NCCC2=CC=C(C=C2)S(=O)(=O)NC(=O)NC2CCC(C)CC2)C1=O	7.657
COCCOC[C@H](CC1(CCCC1)C(=O)N[C@H]1CC[C@H](CC1)C(O)=O)C(=O)OC1=CC2=C(CCC2)C=C1	7.3445
CC(C)C1CC[C@@H](CC1)C(=O)N[C@H](CC1=CC=CC=C1)C(O)=O	6.7911
OC(=O)C(CC(=O)N1CC2CCCCC2C1)CC1=CC=CC=C1	6.7525
CN(C)C1=NC(=NC(=N1)N(C)C)N(C)C	6.7024
CC1=CC=C(C=C1)S(=O)(=O)NC(=O)NN1CC2CCCC2C1	6.5387
COC1=C(C=C(Cl)C=C1)C(=O)NCCC1=CC=C(C=C1)S(=O)(=O)NC(=O)NC1CCCCC1	6.28
COC1=C(CC2=CN(C)C3=C2C=C(NC(=O)OC2CCCC2)C=C3)C=CC(=C1)C(=O)NS(=O)(=O)C1=CC=CC=C1C	6.2556
CCCN(CCC)CCC1=C2CC(=O)NC2=CC=C1	6.061
CC(C)NCC(O)COC1=CC=C(CCOCC2CC2)C=C1	5.948
CCCCCCCCCCCCCCCCCCCCCCO	5.9243
O=C1CC2(CCCC2)CC(=O)N1CCCCN1CCN(CC1)C1=NC=CC=N1	5.8772
CCOC1=CC(N)=C(C=C1C(=O)NC1CCN(CC2CCC=CC2)CC1)[N+]([O-])=O	5.8503
FC1=CC=C(C=C1)C(CCCN1CCC(CC1)N1C(=O)NC2=CC=CC=C12)C1=CC=C(F)C=C1	5.8017
COC1=CC(CC2=CN=C(N)N=C2N)=CC(OC)=C1OC	5.701
COC1=CC(NCC2=C(C)C3=C(C=C2)N=C(N)N=C3N)=CC(OC)=C1OC	5.6809
CCN(CCCC1=CC=CC=C1)CCCC1=CC=CC=C1	5.6401
NC(=N)C1=CC=C(OCCCCCOC2=CC=C(C=C2)C(N)=N)C=C1	5.6056
CCC1(CCC(C)C)C(=O)NC(=O)NC1=O	5.4584
CN(C)CCC1=CNC2=C1C=C(CN1C=NC=N1)C=C2	5.4311
CC(C)CC1=CC=C(C=C1)C(C)C(O)=O	5.4135
CC(C)CC(N(C)C)C1(CCC1)C1=CC=C(Cl)C=C1	5.3913
CC(C)(C)C(=O)OCOP(=O)(COCCN1C=NC2=C(N)N=CN=C12)OCOC(=O)C(C)(C)C	5.3834
CC(C)[C@H](N)C(=O)OCCOCN1C=NC2=C1NC(N)=NC2=O	5.3733
CN1N=C(C(=O)NC2CC3CCCC(C2)N3C)C2=CC=CC=C12	5.3389
CN1[C@H]2CC[C@@H]1C[C@@H](C2)OC(=O)C(CO)C1=CC=CC=C1	5.3217
CC(C)COCC(CN(CC1=CC=CC=C1)C1=CC=CC=C1)N1CCCC1	5.2783
CCN1N=NN(CCN2CCC(COC)(CC2)N(C(=O)CC)C2=CC=CC=C2)C1=O	5.2659
CC(C)NCC(O)COC1=CC=C(COCCOC(C)C)C=C1	5.2609
CN1[C@H]2CC[C@@H]1C[C@@H](C2)OC(=O)[C@H](CO)C1=CC=CC=C1	5.2472
OC1=C(C=C(Cl)C=C1)C(=O)NC1=C(Cl)C=C(C=C1)[N+]([O-])=O	5.2426
CCCCNC(=O)NS(=O)(=O)C1=CC=C(C)C=C1	5.167
ClC1=CC(Cl)=C(COC(CN2C=CN=C2)C2=C(Cl)C=C(Cl)C=C2)C=C1	5.1454
CC1=CC=C(C=C1)C(=O)C1=CC(=C(O)C(O)=C1)[N+]([O-])=O	5.092
COC1=CC2=NNN=C2C=C1C(=O)NCC1CCCN1CC=C	5.0598
CCCCCCCN(CC)CCCC(O)C1=CC=C(NS(C)(=O)=O)C=C1	5.0534
CCCNCC(O)COC1=CC=CC=C1C(=O)CCC1=CC=CC=C1	4.9973
N[C@@H](CC1=CN=CN1)C(O)=O	4.9548
CN(CC1=CC=C(C=C1)C(C)(C)C)CC1=CC=CC2=CC=CC=C12	4.9507
COCCC1=CC=C(OCC(O)CNC(C)C)C=C1	4.9426
CCC1=NN(CCCN2CCN(CC2)C2=CC(Cl)=CC=C2)C(=O)N1CCOC1=CC=CC=C1	4.9373
CCCCC1(CC)C(=O)NC(=O)NC1=O	4.9305
CCCN(CCC1=CC=CS1)C1CCC2=C(C1)C=CC=C2O	4.9178
CC(=O)OCC(CCN1C=NC2=CN=C(N)N=C12)COC(C)=O	4.9138
CO[C@H]1CN(CCCOC2=CC=C(F)C=C2)CC[C@H]1NC(=O)C1=CC(Cl)=C(N)C=C1OC	4.9129
NC(=N)N1CCC2=CC=CC=C2C1	4.8682
ClC1=CC=C(COC(CN2C=CN=C2)C2=C(Cl)C=C(Cl)C=C2)C=C1	4.8625
CC1=CN=C(C=N1)C(=O)NCCC1=CC=C(C=C1)S(=O)(=O)NC(=O)NC1CCCCC1	4.861
N[C@@H](CC1=CC=CC=C1)C(O)=O	4.8219
CN(CC=CC1=CC=CC=C1)CC1=CC=CC2=CC=CC=C12	4.8123
COC1=CC(C(O)C(C)N)=C(OC)C=C1	4.7948
COC1=C(OC)C=C2C(N)=NC(=NC2=C1)N(C)CCCNC(=O)C1CCCO1	4.7908
CC(C)CC1(CC=C)C(=O)NC(=O)NC1=O	4.7818
FC1=CC=C(C=C1)C(=O)CCCN1CCC(=CC1)N1C(=O)NC2=CC=CC=C12	4.7367
COC1=CC=C(C=C1)C(=O)NC1=CC=CC=C1CCC1CCCCN1C	4.7264
O=C1CCC2=C(N1)C=CC(OCCCCC1=NN=NN1C1CCCCC1)=C2	4.7135
CN1CCC[C@@H]1CCO[C@](C)(C1=CC=CC=C1)C1=CC=C(Cl)C=C1	4.7082
C(C(C1CCCCC1)C1CCCCC1)C1CCCCN1	4.6999
CC1=CC(=O)N(O)C(=C1)C1CCCCC1	4.6909
CCCC(=O)O[C@H](COC(=O)CC)COP(O)(=O)OC[C@H](N)C(O)=O	4.671
CC1=CC=C(C=C1)S(=O)(=O)NC(=O)NN1CCCCCC1	4.6597
COC1=CC2=C(C=CC=C2CCNC(C)=O)C=C1	4.6382
CC(C)NCC(O)C1=CC(O)=C(O)C=C1	4.6327
ClC1=C(COC(CN2C=CN=C2)C2=C(Cl)C=C(Cl)C=C2)C=CS1	4.6304
CC1=C(C)N=C(NS(=O)(=O)C2=CC=C(N)C=C2)O1	4.6169
CC(C=CC1=C(C)CCCC1(C)C)=CC=CC(C)=CC(O)=O	4.6135
COC1=CC2=C(NC=C2CCNC(C)=O)C=C1	4.5993
CC[C@@H](N1CCCC1=O)C(N)=O	4.5956
CC(C)NCC(O)COC1=CC=CC=C1CC=C	4.5892
COC1=C(OC)C=C2C(N)=NC(=NC2=C1)N1CCN(CC1)C(=O)C1CCCO1	4.5855
CCCN(CCC)S(=O)(=O)C1=CC=C(C=C1)C(O)=O	4.5747
N[C@@H](CC1=CNC2=CC=CC=C12)C(O)=O	4.5676
COC1=C(OC)C=C2C(N)=NC(=NC2=C1)N1CCN(CC1)C(=O)C1=CC=CO1	4.5607
COC(=O)CCC1=CC=C(OCC(O)CNC(C)C)C=C1	4.559
CC(C)NCC(O)COC1=CC=CC2=CC=CC=C12	4.5575
NC1=CC=C(C=C1)S(=O)(=O)NC1=CC=NN1C1=CC=CC=C1	4.5554
CCCC(C)C1(CC=C)C(=O)NC(=O)NC1=O	4.5553
CCN(CC)CCCN(C1CC2=CC=CC=C2C1)C1=CC=CC=C1	4.5518
N[C@@H](CCC(O)=O)C(O)=O	4.5412
O=C(NC1=CC2=C(C=C1)C(=O)C=C(O2)C1=NNN=N1)C1=CC=C(OCCCCC2=CC=CC=C2)C=C1	4.5399
COC1=CC2=C(C=C1)C=C(CCC(C)=O)C=C2	4.5388
CCC1=CN=C(CCOC2=CC=C(CC3SC(=O)NC3=O)C=C2)C=C1	4.5281
CCC(=C(CC)C1=CC=C(O)C=C1)C1=CC=C(O)C=C1	4.5246
CC(C)[C@@H]1CC[C@@H](C)C[C@H]1O	4.5244
CC(C)(N)CC1=CC=CC=C1	4.519
CN1CCCC(CC2C3=CC=CC=C3SC3=CC=CC=C23)C1	4.5063
C(C1=NCCN1)C1=CC=CC2=CC=CC=C12	4.5059
COC(=O)NC1=NC2=C(N1)C=C(C=C2)C(=O)C1=CC=CC=C1	4.4965
CCN(CC)CCNC(=O)C1=CC(Cl)=C(N)C=C1OC	4.4815
CC(N)CC1=CC=CC=C1	4.4686
CC(CCC1=CC=CC=C1)NCC(O)C1=CC(C(N)=O)=C(O)C=C1	4.46
COC1=NC=CN=C1NS(=O)(=O)C1=CC=C(N)C=C1	4.4456
CCC(=O)N(C1=CC=CC=C1)C1(CCN(CCC(=O)OC)CC1)C(=O)OC	4.438
CC(C)N(CC[C@H](C1=CC=CC=C1)C1=C(O)C=CC(C)=C1)C(C)C	4.4327
C[C@H](C1=CNC=N1)C1=C(C)C(C)=CC=C1	4.4282
CC(C)(C)NCC(O)C1=CC(Cl)=C(N)C(Cl)=C1	4.4149
CCN1C(=O)NC(C1=O)C1=CC=CC=C1	4.411
CCOC(=O)C1(CCN(CCC2=CC=C(N)C=C2)CC1)C1=CC=CC=C1	4.3966
CC(=O)C1=CC=C(C=C1)S(=O)(=O)NC(=O)NC1CCCCC1	4.395
CC(C)NCC(O)COC1=CC=CC2=C1C=CN2	4.3921
O=C(CCCC1=CC=CC=C1)OCC(COC(=O)CCCC1=CC=CC=C1)OC(=O)CCCC1=CC=CC=C1	4.3813
COC1=CC=C(C=C1)C(Cl)=C(C1=CC=C(OC)C=C1)C1=CC=C(OC)C=C1	4.3799
OC(=O)CCC1=NC(=C(O1)C1=CC=CC=C1)C1=CC=CC=C1	4.3619
ClC(Cl)C(C1=CC=C(Cl)C=C1)C1=CC=CC=C1Cl	4.3605
CC1=C(OC2=C(C=CC=C2C(=O)OCCN2CCCCC2)C1=O)C1=CC=CC=C1	4.3556
CC(C)NCC(O)COC1=CC=CC=C1OCC=C	4.3528
CCCCOC1=C(N)C=CC(=C1)C(=O)OCCN(CC)CC	4.3329
CC(C)NCC(O)COC1=CC=C(NC(C)=O)C=C1	4.3306
CCC(NC(C)C)C(O)C1=CC(O)=C(O)C=C1	4.3301
C[C@H](CN1C=NC2=C1N=CN=C2N)OCP(O)(O)=O	4.2933
CN[C@H]1CC[C@@H](C2=CC(Cl)=C(Cl)C=C2)C2=CC=CC=C12	4.2886
CC(=O)NS(=O)(=O)C1=CC=C(N)C=C1	4.2868
COC[C@@H](NC(C)=O)C(=O)NCC1=CC=CC=C1	4.2712
CCCC(CCC)C(O)=O	4.2703
N[C@@H](CSSC[C@H](N)C(O)=O)C(O)=O	4.2604
CC=C(C(=CC)C1=CC=C(O)C=C1)C1=CC=C(O)C=C1	4.2578
CN(C)CCC(C1=CC=C(Cl)C=C1)C1=CC=CC=N1	4.2572
CN1C(=O)CC(C1=O)C1=CC=CC=C1	4.2564
CCC1(CC)C(=O)NC(=O)N(C)C1=O	4.2557
CC(COC1=CC=CC=C1)N(CCCl)CC1=CC=CC=C1	4.2525
C1=CN(C=N1)C(C1=CC=CC=C1)C1=CC=C(C=C1)C1=CC=CC=C1	4.2453
CCC(C)C1(CC=C)C(=O)NC(=O)NC1=O	4.2429
CNC(C)CCC=C(C)C	4.2383
CC[C@H]1[C@@H](CC2=CN=CN2C)COC1=O	4.232
CCOC(=O)N1C=CN(C)C1=S	4.2193
CCCC(C)C1(CC)C(=O)NC(=O)NC1=O	4.2154
CCCSC1=CC2=C(C=C1)N=C(NC(=O)OC)N2	4.2147
OC(=O)CCCC1=CC=C(C=C1)N(CCCl)CCCl	4.1857
CN(C)CCC(C1=CC=CC=C1)C1=CC=CC=N1	4.1779
FC1=CC=C(C=C1)N1C=C(C2CCN(CCN3CCNC3=O)CC2)C2=C1C=CC(Cl)=C2	4.1777
NC1=C2CCCCC2=NC2=CC=CC=C12	4.1688
CC(C)NCC(O)C1=CC(O)=CC(O)=C1	4.1666
CC(=O)OCC(=O)NCCCOC1=CC=CC(CN2CCCCC2)=C1	4.1621
CCCCCCCCCCCCOCCO	4.1454
CCCCOC1=NC2=CC=CC=C2C(=C1)C(=O)NCCN(CC)CC	4.1434
CCN(CC)CCOC1=CC=C(C=C1)C(=C(Cl)C1=CC=CC=C1)C1=CC=CC=C1	4.1393
COCCOC1=CN=C(NS(=O)(=O)C2=CC=CC=C2)N=C1	4.129
CC1=CC(=CC(C)=C1CC1=NCCN1)C(C)(C)C	4.128
CN(CCOC1=CC=C(CC2SC(=O)NC2=O)C=C1)C1=CC=CC=N1	4.1273
CN1CCC(CC1)OC(C1=CC=CC=C1)C1=CC=CC=C1	4.1235
CCN(CC)CCOC(=O)C1=C(Cl)C=C(N)C=C1	4.1159
CCCCC1=NC(Cl)=C(CO)N1CC1=CC=C(C=C1)C1=CC=CC=C1C1=NNN=N1	4.1151
O=C1N=CN=C2NNC=C12	4.1095
COC1=CC2=C(C=C1)C=C(C=C2)[C@H](C)C(O)=O	4.1047
CC1=CC2=C(N1)C=CC=C2OCC(CNC(C)(C)C)OC(=O)C1=CC=CC=C1	4.0987
N1C2=CC=CC=C2N=C1C1=CSC=N1	4.0878
COC1=C(OC)C=C2C3CC(=O)C(CC(C)C)CN3CCC2=C1	4.0859
NC1=C2NC=NC2=NC=N1	4.0841
CCC(C)C1(CC)C(=O)NC(=O)NC1=O	4.0792
[O-][N+](=O)C1=CC=C(C=C1)C1=CC=C(O1)C=NN1CC(=O)NC1=O	4.0755
CN1C2=C(C=C(Cl)C=C2)C(=NC(O)C1=O)C1=CC=CC=C1	4.0695
COC1=C(OC)C=C(CCNCC(O)COC2=CC=CC(C)=C2)C=C1	4.0662
CN(C(=O)C(Cl)Cl)C1=CC=C(OC(=O)C2=CC=CO2)C=C1	4.0599
NC1=CC=C(C=C1)C(=O)NCC(O)=O	4.0555
CC(NC(C)(C)C)C(=O)C1=CC(Cl)=CC=C1	4.055
CN[C@@H](C)CC1=CC=CC=C1	4.0441
COC1=C(OC)C=C(CC2=NC=CC3=CC(OC)=C(OC)C=C23)C=C1	4.0407
CCOC(=O)CCCCCCCCC(C)C1=CC=CC=C1I	4.0284
C[C@@H](CC1=CC=CC=C1)N(C)CC1=CC=CC=C1	4.0265
NC1=CC=C(C=C1)S(=O)(=O)NC1=NC=CC=N1	4.0196
CCC1(NC(=O)N(C)C1=O)C1=CC=CC=C1	4.0159
CC1=CN([C@@H]2O[C@H](CO)C=C2)C(=O)NC1=O	4.0115
NC1=CC(Cl)=C(NC2=NCCN2)C(Cl)=C1	3.9983
NC1=NC(N)=C2N=C(C(N)=NC2=N1)C1=CC=CC=C1	3.9959
OC(=O)C1=CC=CC=C1OC(=O)C1=CC=CC=C1O	3.9916
ClC1=CC=CC(=C1)N1CCN(CCCN2N=C3C=CC=CN3C2=O)CC1	3.9883
ClC1=CC=C(S1)C(=O)NC[C@H]1CN(C(=O)O1)C1=CC=C(C=C1)N1CCOCC1=O	3.9842
CC(CCC1=CC=C(O)C=C1)NCCC1=CC(O)=C(O)C=C1	3.9783
S=C1N=CNC2=C1NC=N2	3.9627
COC1=CC=C(CCN2CCC(CC2)NC2=NC3=CC=CC=C3N2CC2=CC=C(F)C=C2)C=C1	3.9599
CC(C)NCC(O)COC1=C(C)C(C)=C(OC(C)=O)C(C)=C1	3.9588
C(N(CC1=CC=CC=C1)C1=CC=CC=C1)C1=NCCN1	3.9578
NC1=NC(=S)C2=C(N1)N=CN2	3.9538
CC(C)C[C@H](N)C(O)=O	3.9532
CC(C)NC(=O)NS(=O)(=O)C1=C(NC2=CC=CC(C)=C2)C=CN=C1	3.9437
CN1C=NC2=C1C(=O)NC(=O)N2C	3.9378
OC(=O)CCCN1CCC(CC1)OC(C1=CC=C(Cl)C=C1)C1=CC=CC=N1	3.937
CN1C=NC2=C1C(=O)N(CCCCC(C)=O)C(=O)N2C	3.9279
C[C@H](N)CC1=CC=CC=C1	3.9224
C(C=CC1=CC=CC=C1)N1CCN(CC1)C(C1=CC=CC=C1)C1=CC=CC=C1	3.9128
NC1=CC=C(C=C1)S(=O)(=O)NC1=CC=CC=N1	3.9055
C(C1=NCCN1)C1=CC=CC=C1	3.8771
CN1CCN(CC2=CC=C(C=C2)C(=O)NC2=CC(NC3=NC=CC(=N3)C3=CN=CC=C3)=C(C)C=C2)CC1	3.87
N[C@@H]1CONC1=O	3.8665
CC1=CC=C(C=C1)N(CC1=NCCN1)C1=CC(O)=CC=C1	3.8642
OC1=C(CC2=C(O)C3=C(OC2=O)C=CC=C3)C(=O)OC2=C1C=CC=C2	3.8597
COC1=CC(O)=C(C=C1)C(=O)C1=CC=CC=C1	3.852
BrC1=C(NC2=NCCN2)C=CC2=NC=CN=C12	3.8374
CCN(CC)CCOC(=O)C1(CCCCC1)C1CCCCC1	3.8337
CC(C)NCC(O)C1=CC=C(NS(C)(=O)=O)C=C1	3.8223
CC(C(O)=O)C1=CC=C(S1)C(=O)C1=CC=CC=C1	3.8159
CN1CCCC1C1=CN=CC=C1	3.8105
CCN(CC)CC1=C(O)C=CC(NC2=C3C=CC(Cl)=CC3=NC=C2)=C1	3.8102
CC(C)(C)C1=CC=C(CN2CCN(CC2)C(C2=CC=CC=C2)C2=CC=C(Cl)C=C2)C=C1	3.8053
C1CN2C[C@@H](N=C2S1)C1=CC=CC=C1	3.7974
CCCC1=NC2=C(C=C(C=C2C)C2=NC3=CC=CC=C3N2C)N1CC1=CC=C(C=C1)C1=CC=CC=C1C(O)=O	3.7869
CN(C)CCN(CC1=CC=CC=C1)C1=CC=CC=N1	3.7777
NC12CC3CC(CC(C3)C1)C2	3.7696
CCOC(=O)C1(CCN(C)CC1)C1=CC=CC=C1	3.768
ClC1=CC=CC(Cl)=C1NC1=NCCN1	3.7435
CN(C)CCN(CC1=CC=C(Cl)C=C1)C1=CC=CC=N1	3.7364
CCN(CCO)CCCC(C)NC1=C2C=CC(Cl)=CC2=NC=C1	3.7274
CCSC1=CC2=C(SC3=CC=CC=C3N2CCCN2CCN(C)CC2)C=C1	3.7204
CCC1=C(C(N)=NC(N)=N1)C1=CC=C(Cl)C=C1	3.7158
CC(C)OC(=O)C(C)(C)OC1=CC=C(C=C1)C(=O)C1=CC=C(Cl)C=C1	3.7
CC(C)(C)NCC(O)C1=CC(O)=CC(O)=C1	3.683
COC1=CC=C(C=C1)C1C(=O)C2=CC=CC=C2C1=O	3.6757
CCCN1C2=C(NC=N2)C(=O)NC1=O	3.6597
OC(=O)CCCCC1CCSS1	3.6596
NC1=CC(O)=C(C=C1)C(O)=O	3.6575
CCN(C(=O)C=CC)C1=CC=CC=C1C	3.6561
CC(C(O)=O)C1=CC(=CC=C1)C(=O)C1=CC=CC=C1	3.6541
OC(CCN1CCCC1)(C1CCCCC1)C1=CC=CC=C1	3.6539
OC(=O)CN(CCN(CC(O)=O)CC(O)=O)CC(O)=O	3.6529
CC(N)C(=O)NC1=C(C)C=CC=C1C	3.6507
CC(C)C1(CC=C)C(=O)NC(=O)NC1=O	3.6424
O[C@@H]1CNC(C1)C(O)=O	3.6252
OC1(CCN(CCCC(=O)C2=CC=C(F)C=C2)CC1)C1=CC=C(Cl)C=C1	3.6228
CCN1CCCC1CNC(=O)C1=CC(=C(N)C=C1OC)S(=O)(=O)CC	3.6183
CNC(C)CC1CCCCC1	3.6151
CN1CCC[C@@H]1CC1=CNC2=C1C=C(CCS(=O)(=O)C1=CC=CC=C1)C=C2	3.6081
NC(CCC(O)=O)C=C	3.5995
CC1=CC(OCC2CNC(=O)O2)=CC(C)=C1	3.5949
NC(=O)C1=CC=CC=C1O	3.5896
CCCCOC1=CC=C(C=C1)C(=O)CCN1CCCCC1	3.5827
CC1=CC(OCCCC(C)(C)C(O)=O)=C(C)C=C1	3.5725
CCN(CC)C(=O)N1CCN(C)CC1	3.5702
CCOC(=O)C1=CN=CN1[C@H](C)C1=CC=CC=C1	3.5675
CCN1CCCC1CNC(=O)C1=C(OC)C=CC(=C1)S(N)(=O)=O	3.5621
ClC1=CC2=C(OC(=O)N2)C=C1	3.5614
CCN(CC1=CC=NC=C1)C(=O)C(CO)C1=CC=CC=C1	3.5501
O=C1N(C2CCC(=O)NC2=O)C(=O)C2=CC=CC=C12	3.5307
CC(C)(OC1=CC=C(CCNC(=O)C2=CC=C(Cl)C=C2)C=C1)C(O)=O	3.5243
CC(OC1=C(Cl)C=CC=C1Cl)C1=NCCN1	3.5195
CC1CC(CC(C)(C)C1)OC(=O)C(O)C1=CC=CC=C1	3.5093
OC(=O)COCCN1CCN(CC1)C(C1=CC=CC=C1)C1=CC=C(Cl)C=C1	3.4952
CCCOC1=C(N)C=C(C=C1)C(=O)OCCN(CC)CC	3.4926
CN(C)CCOC(C1=CC=CC=C1)C1=CC=CC=C1	3.4886
COC(=O)C(C1CCCCN1)C1=CC=CC=C1	3.488
CCCN[C@H]1CCC2=C(C1)SC(N)=N2	3.4868
FC1=CC=C(C=C1)C(N1CCN(CC1)C1=NC(NCC=C)=NC(NCC=C)=N1)C1=CC=C(F)C=C1	3.4866
CCCN1CCCC[C@H]1C(=O)NC1=C(C)C=CC=C1C	3.468
COC1=CC=C(CN(CCN(C)C)C2=NC=CC=C2)C=C1	3.4624
CC(=O)OC1=CC=CC=C1C(O)=O	3.455
OC(=O)CCCCCCCC(O)=O	3.4544
CN1C(=O)OC(C)(C)C1=O	3.45
CCCCN1CCCCC1C(=O)NC1=C(C)C=CC=C1C	3.4483
OC1=C2N=CC=CC2=C(C=C1)[N+]([O-])=O	3.4432
CCC1(C)OC(=O)N(C)C1=O	3.4358
CC(CNC1CCCCC1)OC(=O)C1=CC=CC=C1	3.4185
CC1=CC(=O)C2=CC=CC=C2C1=O	3.406
CCC1=C(C)NC2=C1C(=O)C(CN1CCOCC1)CC2	3.392
O=C(OCC1=CC=CC=C1)C1=CC=CC=C1	3.3773
CC1=CC(CN2CCN(CC2)C(C2=CC=CC=C2)C2=CC=C(Cl)C=C2)=CC=C1	3.3751
CCN(CC)CC(=O)NC1=C(C)C=CC=C1C	3.3688
CS(=O)(=O)C1=CC(Cl)=C(C=C1)C(=O)NC1=CC=C(Cl)C(=C1)C1=CC=CC=N1	3.3675
OC(CCN1CCCCC1)(C1CC2CC1C=C2)C1=CC=CC=C1	3.3635
O=C(C1CCCCC1)N1CC2N(CCC3=CC=CC=C23)C(=O)C1	3.3627
CCCNC(C)C(=O)NC1=CC=CC=C1C	3.3422
CC(C(O)=O)C1=CC2=C(C=C1)C1=C(N2)C=CC(Cl)=C1	3.3392
CC(C)C1=CC=CC(C(C)C)=C1O	3.3379
COC1=CC=CC=C1OCC(O)CN1CCN(CC(=O)NC2=C(C)C=CC=C2C)CC1	3.3373
CN1CCCCC1C(=O)NC1=C(C)C=CC=C1C	3.3366
CCC1(C)CC(=O)NC1=O	3.3363
[O-][N+](=O)C1=CC=C(O1)C=NN1CCOC1=O	3.3153
OC(=O)C1=CC=CC=C1O	3.3147
CC1=C(C)C(NC2=CC=CC=C2C(O)=O)=CC=C1	3.3142
CN1C2=C(NC=N2)C(=O)N(C)C1=O	3.3104
OC(=O)[C@@H]1CCCN1	3.3103
CCCCC1=NN(CC2=CN=C(C=C2)C2=CC=CC=C2C2=NNN=N2)C(CCCC)=N1	3.3095
CCN(CC)CCOC(=O)C1=CC=C(N)C=C1	3.3078
CCCCN1CCCC[C@H]1C(=O)NC1=C(C)C=CC=C1C	3.307
CC(N)C12CC3CC(CC(C3)C1)C2	3.2932
CCCCC1=NC2(CCCC2)C(=O)N1CC1=CC=C(C=C1)C1=CC=CC=C1C1=NNN=N1	3.2863
CN1C=CNC1=S	3.2765
CC(CC1=CC=C(O)C=C1)NCC(O)C1=CC(O)=CC(O)=C1	3.2612
CCCC(=O)NC1=CC(C(C)=O)=C(OCC(O)CNC(C)C)C=C1	3.2558
CC1=NC=C(N1CCO)[N+]([O-])=O	3.2451
OC(CCN1CCCCC1)(C1CCCCC1)C1=CC=CC=C1	3.2362
CN1CCN(CC1)C(C1=CC=CC=C1)C1=CC=CC=C1	3.2288
CSC1=CC=C(C=C1)C(=O)C1=C(C)NC(=O)N1	3.227
CN(C)CCOC(C1=CC=CC=C1)C1=CC=CC=C1C	3.2248
OC(CCN1CCCCC1)(C1CCCC1)C1=CC=CC=C1	3.2185
NC1=CC=NC=C1	3.2158
CC1=CNN=C1	3.1896
COC1=C(OC)C=C2C(N)=NC(=NC2=C1)N1CCN(CC1)C(=O)C1COC2=CC=CC=C2O1	3.1822
CCC1=NC=CC(=C1)C(N)=S	3.1661
CCN(CC)CCNC(=O)C1=CC=C(N)C=C1	3.166
COC1=CC=C(CC(C)NCC(O)C2=CC(NC=O)=C(O)C=C2)C=C1	3.165
CCOCCN1C(=NC2=CC=CC=C12)N1CCCN(C)CC1	3.1551
CSC1=CC2=C(SC3=CC=CC=C3N2CCC2CCCCN2C)C=C1	3.1429
N[C@@H]1CC1C1=CC=CC=C1	3.136
C[C@H](N)C(O)=O	3.1245
OC(=O)P(O)(O)=O	3.1094
CCN(CC)C(C)C(=O)C1=CC=CC=C1	3.1013
CNC1(C)C2CCC(C2)C1(C)C	3.1002
OCCCC([O-])=O	3.0886
CC1=CC(OCC(O)CNC(C)(C)C)=C(Cl)C=C1	3.0855
CN1C2=C(C=C(Cl)C=C2)C(=NCC1=O)C1=CC=CC=C1	3.0848
OC(=O)CCC(O)=O	3.0847
CCC1(C(=O)NC(=O)N(C)C1=O)C1=CC=CC=C1	3.0826
CN1C(=O)CC(C)(C1=O)C1=CC=CC=C1	3.0796
CCC1(CC)C(=O)NCC(C)C1=O	3.0788
CN1C(CC(O)=O)=CC=C1C(=O)C1=CC=C(C)C=C1	3.0748
CC(C)[C@H](N)C(O)=O	3.0747
CCOC(=O)C(C)(C)OC1=CC=C(Cl)C=C1	3.073
NC1=NC(N)=C(N=N1)C1=C(Cl)C(Cl)=CC=C1	3.0561
OC(=O)CC1=CC=CC=C1NC1=C(Cl)C=CC=C1Cl	3.0542
NC1=CC(=NC(N)=[N+]1[O-])N1CCCCC1	3.052
CN1CCN2C(C1)C1=CC=CC=C1CC1=CC=CC=C21	3.0512
OC(CCCN1CCCCC1)(C1=CC=CC=C1)C1=CC=CC=C1	3.0364
O=C1C(C(=O)C2=CC=CC=C12)C1=CC=CC=C1	3.0293
CCC1(CCC(=O)NC1=O)C1=CC=C(N)C=C1	3.0264
CCC1C2=CC(OC)=C(OC)C=C2C(=NN=C1C)C1=CC(OC)=C(OC)C=C1	3.0217
OCC(O)COC1=CC=C(Cl)C=C1	3.021
CCN(CC)CCCC(C)NC1=C2C=CC(Cl)=CC2=NC=C1	3.014
COC1=C(OCCCN2CCOCC2)C=C2C(NC3=CC(Cl)=C(F)C=C3)=NC=NC2=C1	3.0046
CN1N(C(=O)C=C1C)C1=CC=CC=C1	2.9964
NC1=CC(C(O)=O)=C(O)C=C1	2.9921
CCOC(=O)NC1=C(N)C=C(NCC2=CC=C(F)C=C2)C=C1	2.9857
ClC1=C(CCN2CCN(CC2)C2=NSC3=CC=CC=C23)C=C2CC(=O)NC2=C1	2.984
OC1=C(Cl)C=C(Cl)C2=C1N=CC=C2	2.9785
CCOC(=O)C1=CC=C(N)C=C1	2.9674
NC1=NC(=O)C2=C(N1)N(COCCO)C=N2	2.9585
ClC1=C(NC2=NCCN2)C2=NSN=C2C=C1	2.9531
CC(=O)NC1=CC=C(O)C=C1	2.9383
CN(C)CCOC(C1=CC=C(Cl)C=C1)C1=CC=CC=N1	2.933
ClC1=CC=C(C=C1)C(=O)NCCN1CCOCC1	2.9176
CC1=CC(=C(O)C(C)=C1CC1=NCCN1)C(C)(C)C	2.908
CN1CCN(CC1)C1=NC2=CC(Cl)=CC=C2NC2=CC=CC=C12	2.8934
O[Bi]1OC(=O)C2=CC=CC=C2O1	2.8838
CC1=CC(=NO1)C(=O)NNCC1=CC=CC=C1	2.8838
NCC1(CC(O)=O)CCCCC1	2.8533
OC(=O)C1=CC=C(NC(=O)[C@H](CC2=CC=C(O)C=C2)NC(=O)C2=CC=CC=C2)C=C1	2.8411
CC(C(O)=O)C1=CC(OC2=CC=CC=C2)=CC=C1	2.8189
CC[C@H](C)[C@H](N)C(O)=O	2.7924
CN1CCN2C(C1)C1=CC=CC=C1CC1=C2N=CC=C1	2.7413
CC(C)NC1=C(N=CC=C1)N1CCN(CC1)C(=O)C1=CC2=C(N1)C=CC(NS(C)(=O)=O)=C2	2.7251
CCC1(C(=O)NC(=O)NC1=O)C1=CC=CC=C1	2.7218
OC1=CC=CC=C1	2.7195
NC(=O)C1=NC=CN=C1	2.7092
ClC1=CC=C2N=C3NC(=O)CN3CC2=C1Cl	2.6664
CC1=C(Cl)C(NC2=CC=CC=C2C(O)=O)=C(Cl)C=C1	2.665
OC1=CC=C(OCC2=CC=CC=C2)C=C1	2.6436
NC1=CC=C(C=C1)S(N)(=O)=O	2.5847
CCCC1=CC(=O)NC(=S)N1	2.5686
CC(C)(C(=O)C1=CN=CC=C1)C1=CN=CC=C1	2.5476
OC(=O)C1=CN=CC=C1	2.5144
CN1C(=O)NC(=O)C(C)(C1=O)C1=CCCCC1	2.4741
CCCOC1=C(C=C(C=C1)S(=O)(=O)NCCC1CCCN1C)C1=NC(=O)C2=C(N1)C(CCC)=NN2C	2.4651
NC1=CC=CC2=C1CN(C1CCC(=O)NC1=O)C2=O	2.4402
CCC(=C)C(=O)C1=C(Cl)C(Cl)=C(OCC(O)=O)C=C1	2.4091
CCC1(C(=O)NC(=O)NC1=O)C1=CCCCCC1	2.3858
CCC1(CCC(=O)NC1=O)C1=CC=CC=C1	2.3774
OCC1=CC=CC=C1	2.3389
CC1=CC=CC=C1N1CCN(CCC2=NN=C3CCCCN23)CC1	2.3216
NC1=CC(=CNC1=O)C1=CC=NC=C1	2.314
C1CNCCN1	2.304
CC(=O)C(O)=O	2.1885
OC(=O)C1CCN2C1=CC=C2C(=O)C1=CC=CC=C1	2.1507
CCOC(=O)CC(SP(=S)(OC)OC)C(=O)OCC	2.1161
CC1=CC(=NN=C1NCCN1CCOCC1)C1=CC=CC=C1	2.0988
COC1=C2OC(=O)C=CC2=CC2=C1OC=C2	2.0963
OC1N=C(C2=CC=CC=C2)C2=C(NC1=O)C=CC(Cl)=C2	2.0811
CC([O-])=O	2.0735
CCCCNC1=CC=C(C=C1)C(=O)OCCOCCOCCOCCOCCOCCOCCOCCOCCOC	2.0558
CC(C)C1=C(OCC2=NCCN2)C=C(C)C=C1	1.9714
CCCCCCCCCC1=CC=C(OCCOCCOCCOCCOCCOCCOCCOCCOCCO)C=C1	1.9135
OC(=O)C1N=C(C2=CC=CC=C2)C2=C(NC1=O)C=CC(Cl)=C2	1.9133
CC(=O)NC[C@H]1CN(C(=O)O1)C1=CC(F)=C(C=C1)N1CCOCC1	1.9014
CC1=NS(=O)(=O)C2=C(N1)C=CC(Cl)=C2	1.8688
CC1C(OCCN1C)C1=CC=CC=C1	1.8681
[O-][N+](=O)C1=CC2=C(NC(=O)CN=C2C2=CC=CC=C2)C=C1	1.8203
CC(=O)NO	1.728
CC1NCCOC1C1=CC=CC=C1	1.6547
CC1=CC2=CC3=C(OC(=O)C=C3C)C(C)=C2O1	1.5526
CCN1C=C(C(O)=O)C(=O)C2=C1N=C(C)C=C2	1.5024
CN1C=CC(=O)C(O)=C1C	1.4429
BrC1=CC2=C(NC(=O)CN=C2C2=CC=CC=N2)C=C1	1.3273
CC1=CC=CN2C(=O)C(=CN=C12)C1=NNN=N1	1.1747
C[C@@H](CN1CC(=O)NC(=O)C1)N1CC(=O)NC(=O)C1	1.0795

## References

[1] Amata E., Pittala V., Marrazzo A., Parenti C., Prezzavento O., Arena E., Nabavi S.M., Salerno L. (2017). Role of the Nrf2/HO-1 axis in bronchopulmonary dysplasia and hyperoxic lung injuries. Clin. Sci..

[2] Pittala V., Salerno L., Romeo G., Acquaviva R., Di Giacomo C., Sorrenti V. (2017). Therapeutic potential of Caffeic Acid Phenethyl Ester (CAPE) in diabetes. Curr. Med. Chem..

[3] Pittala V., Vanella L., Salerno L., Romeo G., Marrazzo A., Di Giacomo C., Sorrenti V. (2017). Effects of polyphenolic derivatives on heme oxygenase-system in metabolic dysfunctions. Curr. Med. Chem..

[4] Salerno L., Pittala V., Romeo G., Modica M.N., Marrazzo A., Siracusa M.A., Sorrenti V., Di Giacomo C., Vanella L., Parayath N.N., Greish K. (2015). Novel imidazole derivatives as heme oxygenase-1 (HO-1) and heme oxygenase-2 (HO-2) inhibitors and their cytotoxic activity in human-derived cancer cell lines. Eur. J. Med. Chem..

[5] Salerno L., Pittala V., Romeo G., Modica M.N., Siracusa M.A., Di Giacomo C., Acquaviva R., Barbagallo I., Tibullo D., Sorrenti V. (2013). Evaluation of novel aryloxyalkyl derivatives of imidazole and 1,2,4-triazole as heme oxygenase-1 (HO-1) inhibitors and their antitumor properties. Bioorg. Med. Chem..

[6] Pittala V., Salerno L., Romeo G., Modica M.N., Siracusa M.A. (2013). A focus on heme oxygenase-1 (HO-1) inhibitors. Curr. Med. Chem..

[7] Sorrenti V., Guccione S., Di Giacomo C., Modica M.N., Pittala V., Acquaviva R., Basile L., Pappalardo M., Salerno L. (2012). Evaluation of Imidazole-based compounds as heme oxygenase-1 inhibitors. Chem. Biol. Drug Des..

[8] Toropova A.P., Toropov A.A., Veselinovic J.B., Miljkovic F.N., Veselinovic A.M. (2014). QSAR models for HEPT derivatives as NNRTI inhibitors based on Monte Carlo method. Eur. J. Med. Chem..

[9] Amata E., Xi H., Colmenarejo G., Gonzalez-Diaz R., Cordon-Obras C., Berlanga M., Manzano P., Erath J., Roncal N.E., Lee P.J., Leed S.E., Rodriguez A., Sciotti R.J., Navarro M., Pollastri M.P. (2016). Identification of "Preferred" human kinase inhibitors for sleeping sickness lead discovery. Are some kinases better than others for inhibitor repurposing?. ACS Infect. Dis..

[10] Diaz R., Luengo-Arratta S.A., Seixas J.D., Amata E., Devine W., Cordon-Obras C., Rojas-Barros D.I., Jimenez E., Ortega F., Crouch S., Colmenarejo G., Fiandor J.M., Martin J.J., Berlanga M., Gonzalez S., Manzano P., Navarro M., Pollastri M.P. (2014). Identification and characterization of hundreds of potent and selective inhibitors of *Trypanosoma brucei* growth from a kinase-targeted library screening campaign. PLoS Negl. Trop. Dis..

[11] Nastasi G., Miceli C., Pittala V., Modica M.N., Prezzavento O., Romeo G., Rescifina A., Marrazzo A., Amata E. (2017). S2RSLDB: a comprehensive manually curated, internet-accessible database of the sigma-2 receptor selective ligands. J. Chemin..

[12] Amata E., Marrazzo A., Dichiara M., Modica M.N., Salerno L., Prezzavento O., Nastasi G., Rescifina A., Romeo G., Pittala V. (2017). Heme oxygenase database (HemeOxDB) and QSAR analysis of isoform 1 inhibitors. ChemMedChem.

[13] Toropova A.P., Toropov A.A., Benfenati E. (2015). CORAL: prediction of binding affinity and efficacy of thyroid hormone receptor ligands. Eur. J. Med. Chem..

[14] Rescifina A., Floresta G., Marrazzo A., Parenti C., Prezzavento O., Nastasi G., Dichiara M., Amata E. (2017). Development of a Sigma-2 receptor affinity filter through a Monte Carlo based QSAR analysis. Eur. J. Pharm. Sci..

[15] Rescifina A., Floresta G., Marrazzo A., Parenti C., Prezzavento O., Nastasi G., Dichiara M., Amata E. (2017). Sigma-2 receptor ligands QSAR model dataset. Data Brief.

