# Supplementary material for: Comprehensive data on a 2D-QSAR model for Heme Oxygenase isoform 1 inhibitors
Source: Data Brief. 2017 Sep 21;15:281–99. doi: 10.1016/j.dib.2017.09.036 (PMC5635207; doi:10.1016/j.dib.2017.09.036)
Supplement: Supplementary file 1 — Transparency document [file mmc1.pdf]

Conflicts of Interest Statement

Manuscript title: Sigma-2 receptor ligands QSAR model dataset

The authors whose names are listed immediately below certify that they have NO affiliations with or involvement in any organization or entity with any financial interest (such as honoraria; educational grants; participation in speakers' bureaus; membership, employment, consultancies, stock ownership, or other equity interest; and expert testimony or patent-licensing arrangements), or non-financial interest (such as personal or professional relationships, affiliations, knowledge or beliefs) in the subject matter or materials discussed in this manuscript.

Author names: Emanuele Amata, Agostino Marrazzo, Maria Dichiara, Maria N. Modica, Loredana Salerno, Orazio Prezzavento, Giovanni Nastasi, Antonio Rescifina, Giuseppe Romeo, Valeria Pittalà.

Catania, 9/7/2017 **Emanuele Amata**

Emanuele Amata

This statement is signed by all the authors to indicate agreement that the above information is true and correct (*a photocopy of this form may be used if there are more than 10 authors*):

Author's name (typed)	Author's signature	Date
